# Contribution to the knowledge of the arthropods community inhabiting the winter-flooded meadows (marcite) of northern Italy

**DOI:** 10.3897/BDJ.9.e57889

**Published:** 2021-01-25

**Authors:** Francesca Della Rocca, Silvia Stefanelli, Elisa Cardarelli, Giuseppe Bogliani, Francesco Bracco

**Affiliations:** 1 Department of Earth and Environmental Sciences, University of Pavia, Via Ferrata 1, Pavia, Italy Department of Earth and Environmental Sciences, University of Pavia, Via Ferrata 1 Pavia Italy; 2 Via Ugo Foscolo 14, 24127, Bergamo, Italy Via Ugo Foscolo 14, 24127 Bergamo Italy; 3 Botanical Garden, University of Pavia, Via S. Epifanio 14, Pavia, Italy Botanical Garden, University of Pavia, Via S. Epifanio 14 Pavia Italy

**Keywords:** arthropods community, threatened habitat, *Lycaena dispar*, *Dolichus halensis*, *Chrysochraon dispar*

## Abstract

**Background:**

Flooded semi-natural grasslands are endangered ecosystems throughout Europe. In Italy, amongst flooded meadows, one special type called “*marcita*” is strongly threatened. It is a stable flooded grassland used to produce green forage even during winter months due to the thermal properties of water coming from springs and fountains that prevent the soil from freezing. To date, some research has been carried out to investigate the role of the *marcita* for ornithological and herpetological communities. However, no comprehensive data on invertebrates inhabiting this particular biotope available. The aim of this study was to characterise the terrestrial entomological community of these typical winter-flooded meadows in northern Italy and, in particular, in six *marcita* fields located in the Ticino Valley Regional Park. We collected data on species richness and diversity of Carabidae, Staphylinidae, Araneae, Lepidoptera and Orthoptera inhabiting *marcita* during the summers of 2014 and 2015 and data on overwintering Coleoptera during the winter of 2014-2015. Amongst the collected species, we identified those highly linked to this habitat.

**New information:**

We found a total of 47 ground beetle species, 35 rove beetle species, 29 spider species, one Lucanidae, 16 butterfly species and 24 grasshopper and cricket species. Most of the species were collected during the summers of 2014 and 2015, while some others were also, or exclusively, overwintering (17 ground beetles, four rove beetles and one Lucanidae) and were collected during the winter of 2014-2015.

*Marcita* fields hosted specialised species and species typical of hygrophilous habitats, amongst which are included the butterfly *Lycaena dispar*, the ground beetle *Dolichus halensis* and the grasshopper *Chrysochraon dispar*. This study represents the first contribution to the knowledge of terrestrial arthropod communities associated with this particular type of winter-irrigated meadow in Europe and confirms the importance of this biotope for invertebrate conservation in agricultural landscapes.

## Introduction

Flooded semi-natural grasslands are highly productive biotopes that support characteristic animal species communities (Prach et al. 1996). Until the early twentieth century, they were widely distributed throughout Europe: in Germany, Belgium, Switzerland and Sweden (Leibundgut and Kohn 2014), in addition to Italy. Later, they fell into disuse and gradually disappeared from the landscape. The main causes of their decline, both in extent and quality, are mechanisation and intensive agriculture which led to a change in land use over the last 50 years (Saarinen et al. 2003, Van Buskirk and Willi 2004). Intensive practices for semi-natural water meadows include higher fertiliser and herbicide applications and the use of modern mowing techniques (Van Buskirk and Willi 2004). This results in eutrophic, structurally poor and homogeneous meadows with negative impacts on diversity, species composition and ecosystem processes (Hans et al. 2020).

In northern Italy and, in particular, in the Po Plain, a typical winter-flooded meadow, the so called “*marcita*” (pronunciation: maarcheeta), is still present, but highly threatened. The *marcita* is a traditional agricultural practice used to produce green forage for domestic animals throughout the year. This agriculture system exploits the thermal properties of water coming from springs and fountains to prevent soil from freezing during periods of intense cold and, due to a network of canals skilfully controlled through sluice gates and earth ridges, a thin layer of water flows smoothly and continuously over the ground during the winter months, allowing a perennial growth of the vegetation (Tomaselli 1960, Ferrari and Lavezzi 1995).

This masterpiece of hydraulic engineering is due to water regimentations that Cistercian monks began in the late thirteenth century by reclaiming the marshes that occupied a large part of the Po Plain. The *marcita* spread consistently from the end of the XVIII century, when modern agriculture brought the development of a more capillary irrigation network (Brown and Redondi 2016).

In the past, the socio-economic role of the *marcita* has been extremely high; farmers could annually carry out 7-8 cuts of green forage, with 3-4 of them collected during the winter. However, since the second World War, the changing agronomic and zootechnical requirements made the *marcita* economically disadvantageous, leading to its progressive conversion into more profitable crops, such as corn, wheat or barley (Gomarasca 2002, Origgi and Guarisco 1992). Therefore, this traditional practice, which, for centuries, was one of the typical and supporting elements of the rural economy of the Po Plain, has now remained as a relict in an agricultural landscape mainly dominated by monocultures (Gomarasca 2002). Today, it survives mainly thanks to the subsidies paid to farmers by some park authorities.

Beyond the importance of the *marcita* as a mixture of cultivation, artwork and historical-cultural elements to preserve, in the last few years, its naturalistic and environmental value has also been recognised (Bove et al. 2017). Indeed, the *marcita*, inserted in a context of intensive agriculture, increases landscape diversity and its aesthetic value. Due to its floristic composition, partly made up of leguminous plants, it has the ability to use atmospheric nitrogen, which reduces or eliminates the need for external inputs, such as fertilisers, agrochemicals and fossil fuels (Bove et al. 2017). Moreover, regulating the water cycle, the *marcita* protects soil from erosion and leaching of nutrients (Bove et al. 2017). The low anthropic pressure, together with the presence of water and vegetation during cold months, allows this habitat to host a rich biodiversity, acting as a refuge and feeding and resting sites for different animal species of conservation interest, especially during the winter season (e.g. amphibians - Gentilli et al. 1997; birds - Casale 2015 and Casale et al. 2016).

Even if some research has been carried out to investigate the role of the *marcita* for ornithological and herpetological communities, to date in Italy, no comprehensive data on invertebrates inhabiting this particular biotope are available. Beyond the conservation interest on their own, many arthropods may play key roles in the maintenance of this ecosystem functioning, feeding on soil invertebrates (Wise et al. 1999), serving as prey for small mammals, amphibians and birds (Holland et al. 2007, Rodríguez and Bustamante 2008) and being natural enemies of crop pests and weeds (Ichihara et al. 2014, Symondson et al. 2002).

Ground beetles (Coleoptera, Carabidae), rove beetles (Coleoptera, Staphylinidae), spiders (Araneae), butterflies (Lepidoptera), grasshoppers and crickets (Orthoptera) are amongst the most common, well-studied and species-rich groups of arthropods in agricultural landscapes and are often used as environmental indicators of human impacts and habitat quality (Báldi and Kisbenedek 1997, Wise et al. 1999, Holland and Luff 2000, Bubová et al. 2015, Marc et al. 1999, Courtney et al. 1982).

The aim of the study was to characterise the terrestrial entomological community of the typical winter-flooded meadows of northern Italy. We quantified species richness and diversity of Carabidae, Staphylinidae, Araneae, Lepidoptera and Orthoptera inhabiting the *marcita* and identified those species highly linked to this habitat.

## Materials and methods

### Study area

The study was conducted in the Ticino Valley Regional Park, in north-western Italy. The Park crosses the most urbanised area of the country and represents an important ecological corridor connecting the Alps to the Po Plain. It is the largest natural area of the entire Po Valley (about 97 km^2^) and encompasses a mosaic of ecosystems, such as riparian woods, patches of primary floodplain forests, large river habitats and wetlands. Amongst the flooded grasslands, the *marcita* extends for a total surface of about 500 ha fragmented into several fields (Bove et al. 2016).

**Figure 1.**
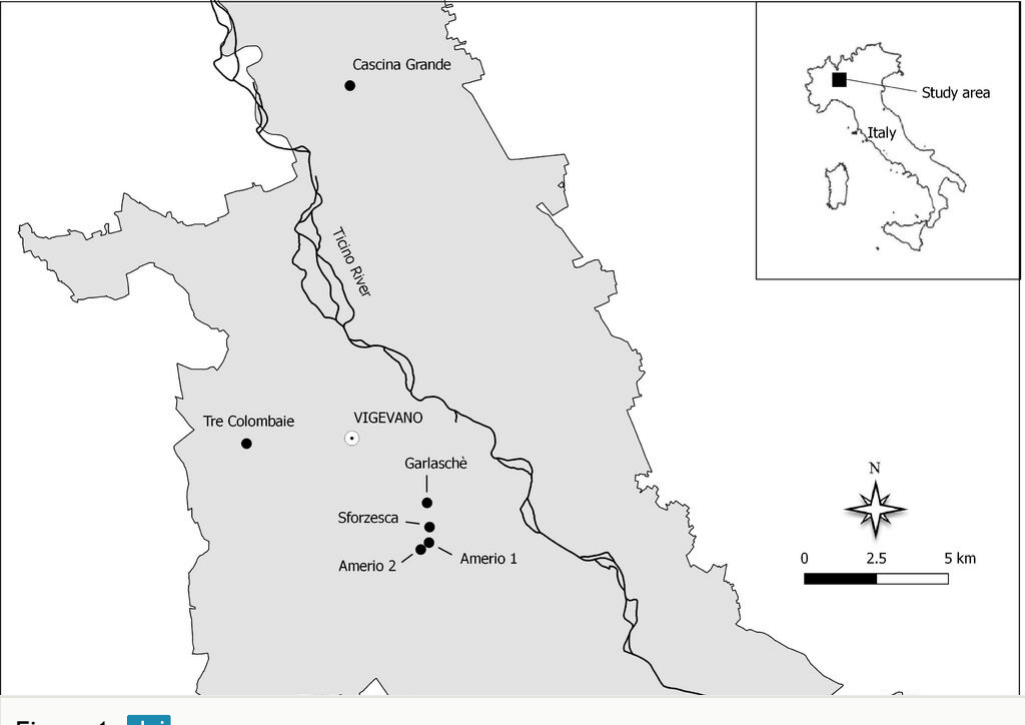
The six *marcita* fields in north-western Italy. In grey, the boundaries of the Ticino Valley Regional Park (Lombardy area) and, in black, the Ticino River.

Six *marcita* fields inside the Park were investigated in 2014 and 2015 for invertebrate assemblages (Fig. 1, Table 1). The fields were mainly named as the near farmhouse or village: Casterno in the Municipality of Robecco sul Naviglio and Tre Colombaie, Sforzesca, Amerio 1, Amerio 2 and Garlaschè in the Municipality of Vigevano.

### Data collection

Ground beetles, rove beetles and spiders were sampled in Sforzesca, Tre Colombaie and Casterno fields from April to October 2014. Six pitfall traps were installed at 10 m intervals along a 50 m linear transect in each field, totalling 18 traps in the whole study area. Once a month, the traps were set, filled with 50 ml of wine vinegar and a drop of detergent, covered with a 10 cm×10 cm wooden roof to prevent flooding and remained active in the field for 10 days. The 10 days of sampling was slightly different amongst the three *marcita* within each month because, during summer, farmers take turns in irrigating the fields. As a consequence, the traps were kept active for 10 days in each field and month, making sure with farmers that irrigation would not take place during those days.

Coleoptera (ground beetles, rove beetles and Lucanidae) were also sampled during November and December 2014 in all six *marcita* fields by actively and opportunistically searching for overwintering species in suitable natural and artificial places along the banks, such as dead woods, barks, stones, wooden boards and earthen banks. Beetles and spiders were preserved in hermetic bottles containing 70% ethanol solution and transported to the laboratory for identification.

Butterflies were sampled in Sforzesca, Tre Colombaie and Casterno fields from April to October 2014. A two-hour long visual census was carried out every two weeks along a 100 m linear transect for a total of 14 sampling dates. Individuals were captured with an entomological net, photographed and then released. During 2015, the butterfly *Lycaena dispar* was opportunistically searched for in all six *marcita* fields.

Grasshoppers and crickets were sampled in all six *marcita* fields from May to September 2015. A two-hour long visual census was carried out monthly along a 100 m linear transect for a total of seven sampling dates. Individuals were sampled by the casual positioning of a transparent plexiglas cylinder, 1 m high and about 30 cm in diameter and by manual collection; specimens were preserved in hermetic bottles containing ethyl acetate and sawdust, placed in a refrigerated bag and transported to the laboratory for identification.

All taxa were identified to the species level by experts (see Acknowledgements) and following the nomenclature of: "Fauna Europaea web project" (De Jong et al. 2014) for ground beetles, rove beetles and butterflies; (World Spider Catalog 2020) for spiders; Massa et al. (2012) for grasshoppers and crickets.

In order to assess the role of *marcita* as a refuge for sensitive species, we selected ground beetles and grasshoppers and crickets as model groups of predatory and herbivorous species. These two groups are well studied (Hůrka 1996, Homburg et al. 2013, Reinhardt et al. 2005, Massa et al. 2012) and information on their ecology and morphology at species level is more exhaustive compared to others taxa. Ground beetles and grasshoppers and crickets were grouped, based on their morpho-ecological features, focusing on their dispersal capability, adult diet and habitat specificity. Specialised species, such as apterous and predatory ones, are known to be spread throughout permanent, undisturbed habitats (Reinhardt et al. 2005, Gobbi and Fontaneto 2008) and their persistence in agricultural landscapes could be highly enhanced by habitat patches with low anthropic pressure (Cardarelli and Bogliani 2014, Giuliano and Bogliani 2018).

For ground beetles, data on wing development and adult diet were derived from Hůrka (1996), Brandmayr et al. (2005), Homburg et al. (2013) and, when not available from literature, from specialist knowledge. The species have been classified as brachypterous (with reduced wings, not suitable for flight), macropterous (with developed wings, suitable for flight) and dimorphic (with both brachypterous and macropterous individuals) and therefore, respectively, with low, high and medium dispersal ability (Brandmayr et al. 2005). As for diet, species were classified as predators, omnivorous and phytophagous. Wing development and diet provide useful information on the level of disturbance and stability of the environment, with wingless and strictly predatory species negatively affected by human impacts (Ribera et al. 2001, Gobbi and Fontaneto 2008). Conversely, mobile, omnivorous species are expected to perform better in disturbed and fragmented habitats due to their major dispersal ability and capacity to use different food resources.

For grasshoppers and crickets, we collected information on dispersal capabilities and habitat specificity. These features are considered important factors in determining species sensitivity to habitat loss and human disturbance, with sedentary and habitat specialist taxa often more susceptible to local extinction events (Reinhardt et al. 2005). Dispersal capabilities were measured using the Mobility Index developed by Reinhardt et al. (2005). Each species was classified into one of three broad mobility classes: sedentary, intermediate dispersers and mobile species. All apterous and brachypterous orthopterans were classified as sedentary, while readily-flying species were assigned as mobile. Furthermore, for species with wing dimorphism (i.e characterised by a solitary or gregarious phase), we considered the most common form observed in the collected sample. Concerning habitat specificity, each species was assessed according to its moisture preferences, following the procedure reported by Reinhardt et al. (2005). We assigned orthopteran species to one of three broad classes: habitat specialists, medium specialised species and generalists. Xerothermophilous and hygrophilous species were classified as habitat specialists, while orthopterans with broad ecological requirements were considered as generalists. The species not treated by Reinhardt et al. (2005) and Marini et al. (2010) were assigned to mobility and habitat specificity classes according to their wing development and their habitat requirements, by gathering this information from Baur et al. (2006) and Massa et al. (2012).

## Checklists

### Araneae

#### Gnaphosidae

38322D97-DB91-560B-B8B5-4701B91B2B75

#### Micaria pulicaria

Sundevall, 1831

101E7D3D-7503-5F21-B5A8-4744817EA686

##### Distribution

Holoarctic species (Isaia et al. 2007). It is distributed in Europe, Turkey, Caucasus, Russia, Central Asia, Mongolia and China (World Spider Catalog 2020). It can be found in mainland Italy and Sardinia (Pantini and Isaia 2019).

##### Notes

It is a widespread species that occurs, at ground level, in a wide variety of locations exposed to the sun: under stones and other debris, at the base of tufts of grass and in leaf litter (Roberts 1995). It is a diurnal hunter and a specialised predator (Isaia et al. 2007)

#### Zelotes longipes

Koch, 1866

070A6C62-31CB-58FA-B3BA-0F7A9EB39697

##### Distribution

It is distributed in Europe, Turkey, Caucasus, Russia (Europe to Far East), Central Asia, Mongolia and China (World Spider Catalog 2020). It can be found in mainland Italy (Pantini and Isaia 2019).

##### Notes

It is widespread in Europe. Lives in dry heathlands, under stones and amongst moss and other vegetation at the base of heather. Adults occur at spring and summer (Roberts 1995). It is a night hunter (Isaia et al. 2007)

#### Linyphiidae

B181B82F-F3A3-56DB-BB2A-37CE995B2061

#### Araeoncus humilis

Blackwall, 1841

C79579F5-A718-5F42-8DAC-094E34EFA3A9

##### Distribution

Palaearctic species (Isaia et al. 2007). It can be found in mainland Italy, Sardinia and Sicily (Pantini and Isaia 2019); in Lombardy, it is reported only in the Province of Bergamo (Isaia et al. 2007).

##### Notes

It is a fairly widespread species, found in a variety of habitats including moss, grass, straw, litter and bark. It is sometimes found in coastal algae litter and sewage filter beds. Adults occur in all seasons (Harvey et al. 2002). It is a weaver spider of simple webs on the ground (Isaia et al. 2007).

#### Bathyphantes gracilis

Blackwall, 1841

9823FB7D-DC33-5873-BA4D-D3237721EA8B

##### Distribution

Holoarctic species (Isaia et al. 2007). It is distributed in North America, Europe, northern Africa, Turkey, Caucasus, Russia (Europe to Far East), Kazakhstan, China, Korea and Japan (World Spider Catalog 2020). It can be found in mainland Italy and Sardinia (Pantini and Isaia 2019).

##### Notes

This ubiquitous spider is common in grasslands and undergrowth of all kinds, including heathlands, woodlands and marshy habitats. Adults can be found at all times of the year, but mostly in the summer and autumn (Harvey et al. 2002). It is a weaver spider of simple webs on the ground (Isaia et al. 2007).

#### Ceratinella brevipes

Westring, 1851

B8479ED3-8B18-5D3C-8ED7-317D3C3C737F

##### Distribution

Sibirico-Europea species (Isaia et al. 2007). It is distributed in Europe, Caucasus andJapan (World Spider Catalog 2020). It can be found in mainland Italy (Pantini and Isaia 2019) and, in Lombardy, it is reported only in the Province of Bergamo (Isaia et al. 2007).

##### Notes

It is found in a variety of situations: in moss and litter layers in various habitats. Adults have been recorded almost year-round, but most frequently from late spring to mid-summer (Harvey et al. 2002). It is a weaver spider of simple webs on the ground (Isaia et al. 2007).

#### Diplostyla concolor

Wider, 1834

A14FC3DA-7DC3-52B4-A1F4-61F8C58226E5

##### Distribution

Holoarctic species (Isaia et al. 2007). It is distributed in North America, Europe, Turkey, Caucasus and Russia (World Spider Catalog 2020). It can be found in mainland Italy and Sardinia (Pantini and Isaia 2019).

##### Notes

It is found in a wide variety of situations, usually at a ground level. The conditions can range from the relative dryness of chalk lowlands to the dampness of marshes. Adults of both sexes are commonly recorded throughout the year (Harvey et al. 2002). It is a weaver spider of simple webs on the ground (Isaia et al. 2007).

#### Erigone dentipalpis

Wider, 1834

FF841556-7C65-5F7B-9C9F-B43555EE8AE2

##### Distribution

Holoarctic species (Isaia et al. 2007). It is distributed in Europe, Turkey, Caucasus, Russia and China (World Spider Catalog 2020). It can be found in mainland Italy, Sardinia and Sicily (Pantini and Isaia 2019).

##### Notes

It is widespread in a range of habitats: particularly in low vegetation, meadows and litter. Adults can be found all throughout the year, with peak numbers in summer (Harvey et al. 2002). It is a weaver spider of simple webs on the ground (Isaia et al. 2007).

#### Gnathonarium dentatum

Wider, 1834

F039F116-B629-59C4-9D0E-FF7E3793C1F7

##### Distribution

Palaearctic species (Isaia et al. 2007). It is distributed in Europe, North Africa, Turkey, Caucasus, Russia to Central Asia, China, Korea and Japan (World Spider Catalog 2020). It can be found in mainland Italy, Sardinia and Sicily (Pantini and Isaia 2019).

##### Notes

It is a wetlands inhabitant frequently found at ground level amongst marsh plants. This species readily colonises disturbed wetland sites and may be found, often in high numbers, in flooded poolside grasslands. Mature specimens of both sexes are present throughout the year (Harvey et al. 2002). It is a weaver spider of simple webs on the ground (Pantini and Isaia 2019).

#### Mermessus trilobatus

Emerton, 1882

1156C142-6E87-51B4-8E32-B58CE6D1ABE5

##### Distribution

It is a North American species. It was introduced to Azores and Europe (World Spider Catalog 2020). It can be found in mainland Italy (Pantini and Isaia 2019).

##### Notes

It is found in meadows, in litter layer of forests, in humid areas and sandy beaches (Harvey et al. 2002).

#### Microlinyphia pusilla

Sundevall, 1830

14574043-AB4E-5A99-BEF0-AF9F669CCD03

##### Distribution

Holoarctic species (Isaia et al. 2007). It is distributed in North America, Europe, North Africa, Turkey, Caucasus, Russia, China, Mongolia and Japan (World Spider Catalog 2020). It can be found in mainland Italy, Sardinia and Sicily (Pantini and Isaia 2019).

##### Notes

It is found in low vegetation and meadows, both dry and wet. It is a "domed" web weaver spider (Isaia et al. 2007).

#### Neriene clathrata

Sundevall, 1830)

924C02EA-3C9F-508F-8025-9691BF1BBAD2

##### Distribution

Holoarctic species (Isaia et al. 2007). It is distributed in North America, Europe, North Africa, Caucasus, Russia (Europe to Far East), Central Asia, China, Korea and Japan (World Spider Catalog 2020). It can be found in mainland Italy, Sardinia and Sicily (Pantini and Isaia 2019).

##### Notes

It occurs on low vegetations and bushes, in a variety of habitats. Adults mature from spring to late autumn (Harvey et al. 2002). It is a "domed" web weaver spider (Isaia et al. 2007).

#### Oedothorax apicatus

Blackwall, 1850

178F1E4A-D9F7-5C12-859D-626D293D6F4C

##### Distribution

Palaearctic species (Isaia et al. 2007). It is distributed in Europe, Turkey, Caucasus, Russia to Central Asia and China (World Spider Catalog 2020). It can be found in mainland Italy (Pantini and Isaia 2019).

##### Notes

It is a widespread species in many open habitats, such as disturbed grasslands, agricultural fields and river pebbles. It is found mainly in low vegetation and in litter. It is a mainly nocturnal active species, which has a low resistance to drying and low temperatures. It is a weaver spider of simple webs on the ground (Isaia et al. 2007). Adults of both sexes have been recorded throughout the year, most often between late spring and mid-summer. Individuals can either overwinter as immature individual or as eggs, depending on the time of reproduction (Harvey et al. 2002).

#### Palliduphantes pallidus

O. Pickard-Cambridge, 1871

2FE48D61-8211-504F-9537-1A100DAD0824

##### Distribution

European species (Isaia et al. 2007). It can be found in mainland Italy (Pantini and Isaia 2019).

##### Notes

It is a troglophile species (Isaia et al. 2007). It is found in a wide variety of habitats, including short grasslands, under stones, moss and litter, on abandoned urban land, in cavities within hollow trees and on dune systems. Adults of both sexes were found throughout the year, with the highest numbers from early to mid-summer (Harvey et al. 2002). It is a "domed" web weaver spider (Isaia et al. 2007).

#### Prinerigone vagans

Audouin, 1826

F1B8E65B-B013-52A6-BF93-5B6499968C9C

##### Distribution

Palaearctic species (Isaia et al. 2007). It can be found in mainland Italy, Sardinia and Sicily (Pantini and Isaia 2019); in Lombardy, it is reported only in the Province of Pavia (Isaia et al. 2007).

##### Notes

It is a hygrophilous species (Isaia et al. 2007). It lives in humid places, including wet grassy meadows, lake shores, gravel pits, sewage filter beds, swamps and in bedding. Adults have been found at most times of the year, but mainly from early to mid-summer and autumn. (Harvey et al. 2002). It is a weaver spider of simple webs on the ground (Isaia et al. 2007).

#### Lycosidae

33F22EC1-1C06-5347-AF11-E6B8694FBD59

#### Alopecosa pulverulenta

Clerck, 1757

0673A2D0-3222-5785-A1CB-F0EF89C39F53

##### Distribution

Palaearctic species (Isaia et al. 2007). It is distributed in Europe, Turkey, Caucasus, Russia, Kazakhstan, China, Korea and Japan (World Spider Catalog 2020). It can be found in mainland Italy, Sardinia and Sicily (Pantini and Isaia 2019).

##### Notes

It is a nocturnal hunter that is especially found in debris. It is a widespread species in open areas of moorland, in grasslands and cultivated land (Isaia et al. 2007).

#### Arctosa leopardus

Sundevall, 1833

B20AEAA1-EEC1-52B1-A26D-60E7070A0102

##### Distribution

Asiatic-European species (Isaia et al. 2007). It is distributed in Europe, Turkey, Caucasus and Russia to Central Asia (World Spider Catalog 2020). It can be found in mainland Italy, Sardinia and Sicily (Pantini and Isaia 2019).

##### Notes

It lives amongst moss and debris in swampy areas. It is a nocturnal hunter whether it weaves tubular webs or runs outdoors (Isaia et al. 2007).

#### Pardosa agrestis

Westring, 1861

35D497BA-555F-5DFE-BC8D-83574064D82B

##### Distribution

Palaearctic species (Isaia et al. 2007). It is distributed in Europe, Caucasus, Russia, Central Asia and China (World Spider Catalog 2020). It can be found in mainland Italy (Pantini and Isaia 2019).

##### Notes

It occurs mainly on poorly vegetated clay soils, in clay pits, mudflats and on the banks of estuaries (Roberts 1995). It is a diurnal hunter (Isaia et al. 2007)

#### Pardosa cribrata

Simon, 1876

1B6D47C3-638D-525E-B508-F1B1A9F958CD

##### Distribution

Mediterrean species (Isaia et al. 2007). Southern Europe, Algeria and Iraq (World Spider Catalog 2020). It can be found in mainland Italy and Sardinia (Pantini and Isaia 2019); in Lombardy, it is reported only in the Province of Cremona (Isaia et al. 2007).

##### Notes

It is a hygrophilous species that hunts during the day in low vegetation and detritus (Isaia et al. 2007).

#### Pardosa prativaga

L. Koch, 1870

1E4EB5E9-42B9-5763-AB83-C3D0B36FA9E8

##### Distribution

Sibiric-European species (Isaia et al. 2007). It is distributed in Europe and Russia (World Spider Catalog 2020). It can be found in mainland Italy and Sardinia (Pantini and Isaia 2019).

##### Notes

It is a diurnal hunter species that lives in low vegetation, meadows and debris (Isaia et al. 2007).

#### Pardosa proxima s.l.

C. L. Koch, 1847

C10EFEA8-D205-5D52-8818-8A7593539B59

##### Distribution

Sibiric-European species (Isaia et al. 2007). It is distributed in Macaronesia, northern Africa, southern Europe, Russia, Central Asia and China (World Spider Catalog 2020). It can be found in mainland Italy, Sardinia and Sicily (Pantini and Isaia 2019).

##### Notes

It is a diurnal hunter species that lives in low vegetation, meadows and debris (Isaia et al. 2007).

#### Pirata tenuitarsis

Simon, 1876

6B458136-3E7B-593D-AD93-8164C8BA43E1

##### Distribution

European species (Isaia et al. 2007). It is distributed from Europe to Mongolia (World Spider Catalog 2020). It can be found in mainland Italy and Sardinia (Pantini and Isaia 2019

##### Notes

It occurs in acid bogs, with most records from *Sphagnum* bogs, often in the vicinity of bog pools and wet heathland (Dawson et al. 2020).

#### Piratula hygrophila

Thorell, 1872

8F03A76C-8DA2-507C-BC26-22813B358D37

##### Distribution

It is distributed in Europe, Turkey, Caucasus and Russia to Central Asia (World Spider Catalog 2020). It can be found in mainland Italy (Pantini and Isaia 2019).

##### Notes

Lives in humid and shady habitats: in forests, in peat bogs or on the soil of the riparian zone (Bazelet and Samways 2011, Roberts 1995).

#### Trochosa ruricola

De Geer, 1778

3C2150DA-9E43-529A-8312-B78A8D1730FF

##### Distribution

Holoarctic species (Isaia et al. 2007). It is distributed from Europe to China, Japan and Korea. Introduced to North America, Cuba, Puerto Rico and Bermuda (World Spider Catalog 2020). It can be found in mainland Italy, Sardinia and Sicily.

##### Notes

It lives under stones and detritus and amongst moss in a variety of damp habitats (Roberts 1995).

#### Tetragnathidae

B8F81B0E-38D6-5DD6-89E9-D9D321AEFB63

#### Pachygnatha clercki

Sundevall, 1823

2C277BC2-CF09-5ADE-90A3-53DF9CA79F5E

##### Distribution

Holoarctic species (Isaia et al. 2007). It is distributed in North America, Europe, Caucasus, Russia, Central Asia, China, Korea and Japan (World Spider Catalog 2020). It can be found in mainland Italy and Sardinia (Pantini and Isaia 2019).

##### Notes

It is a hygrophilous species that lives in low vegetation, meadows and litter. It is a night hunter (Isaia et al. 2007).

#### Pachygnatha degeeri

Sundevall, 1830

E4027AD4-8EE9-5760-B997-022788D6CDB9

##### Distribution

Palearctic species (Isaia et al. 2007). It is distributed in Europe, Turkey, Caucasus, Russia to Central Asia and China (World Spider Catalog 2020). It can be found in mainland Italy, Sardinia and Sicily (Pantini and Isaia 2019).

##### Notes

It is a hygrophilous species that lives in low vegetation, meadows and litter. It is a night hunter (Isaia et al. 2007).

#### Thomisidae

F31FE5F0-47EE-5461-A9E9-CB3014343FB1

#### Ozyptila sanctuaria

O. Pickard-Cambridge, 1871

3F1A1B07-D843-5C0B-A1C8-C818D9DC0013

##### Distribution

European species (Isaia et al. 2007). It can be found in mainland Italy, Sardinia and Sicily (Pantini and Isaia 2019).

##### Notes

It is a xerophilous species that lives in the undergrowth, on tufts of grass, sometimes on chalk or marl. It is a night stalker (Roberts 1995, Isaia et al. 2007).

#### Ozyptila simplex

O. Pickard-Cambridge, 1862

999DF375-1805-5013-833F-9DAA28E1AC20

##### Distribution

Sibiric-European species (Isaia et al. 2007). It is distributed in Europe and Turkey (World Spider Catalog 2020). It can be found in mainland Italy (Pantini and Isaia 2019); in Lombardy, it is reported only in the Province of Pavia (Isaia et al. 2007).

##### Notes

It lives amongst the bases of plants and in detritus, usually in sandy habitats. It is sometimes found higher up on low vegetations (Roberts 1995). It is a night stalker (Isaia et al. 2007).

#### Xysticus gallicus

Simon, 1875

44321138-5E00-5D6A-8297-BF078FF32DAC

##### Distribution

Sibiric-European species (Isaia et al. 2007). It is distributed in Europe, Turkey, Caucasus and Iran (World Spider Catalog 2020). It can be found in mainland Italy (Pantini and Isaia 2019); in Lombardy, it is reported only in the Provinces of Bergamo and Sondrio (Isaia et al. 2007).

##### Notes

It lives in the low vegetation in the pine forests or in the rocky moors, under stones and in detritus from the plains to the alpine areas (Harvey et al. 2002). It is a night stalker (Isaia et al. 2007).

#### Xysticus kochi

Thorell, 1872

005B3FC7-E1BA-5288-A019-D8DA6AD315DB

##### Distribution

Sibiric-European species (Isaia et al. 2007). It is distributed in Europe, from the Mediterranean area to Central Asia (World Spider Catalog 2020). It can be found in mainland Italy, Sardinia and Sicily (Pantini and Isaia 2019).

##### Notes

It is common to find it in low vegetation, on bushes and in the undergrowth. The species prefers warm, dry conditions, provided by open and sparsely-vegetated habitats, such as ruderal habitats, dunes and vegetated pebbles. Adults of both sexes are found mainly in May and June, but females can sometimes survive until autumn (Harvey et al. 2002). It is a night stalker (Isaia et al. 2007).

### Coleoptera

#### Carabidae

49E08E6A-31C6-5870-9921-8DDF2C44F7BE

#### Agonum (Olisares) emarginatum

Gyllenhal, 1827

B1D2A410-08C3-5445-B76F-1A9E770E5EE2

##### Distribution

European species (Hůrka 1996). It can be found in mainland Italy (Vigna Taglianti 2005).

##### Notes

Macropterus. Lives on edges of waters and swamps with vegetation, in floodplain forests, on saline habitats; lowlands and hills (Hůrka 1996).

#### Agonum (Agonum) muelleri

Herbst, 1784

7DC4D61C-9540-525C-9C41-623B22EB56FD

##### Distribution

Holoarctic species ranging eastwards to W Siberia (Hůrka 1996). It can be found in mainland Italy (Vigna Taglianti 2005).

##### Notes

Macropterus. Eurytopic species of mostly unshaded habitats: edges of waters with vegetation, swamps, meadows, fields and ruderal habitats; from lowlands to mountains, mostly in hills (Hůrka 1996).

#### Agonum (Olisares) sexpunctatum

Linnaeus, 1758

90A0010E-C9AC-51D4-9057-BF801B3CB966

##### Distribution

Siberic-European. It can be found in mainland Italy and Sardinia; doubtfully present in Sicily (Vigna Taglianti 2010, Vigna Taglianti 2005).

##### Notes

Macropterus. Lives in moderately moist to wet, unshaded habitats: meadows, pastures, water margins with vegetation, moist forest clearings; from lowlands to mountains, mostly in foothills (Hůrka 1996).

#### Amara (Amara) aenea

DeGeer, 1774

510EB687-86E1-5A09-AE5B-13B5936C4A0A

##### Distribution

Palearctic species, introduced into North America (Hůrka 1996). It is widespread throughout Italy, Sicily and Sardinia (Vigna Taglianti 2005).

##### Notes

Macropterus. Eurytopic species of open habitats: fields, steppe and ruderals; from lowlands to mountains (Hůrka 1996).

#### Amara (Amara) familiaris

Duftschmid, 1812

DF6A49E1-186F-502E-AB4A-8C789287B242

##### Distribution

Palearctic species, ranging central Siberia and north Mongolia, introduced into North America (Hůrka 1996). It can be found in mainland Italy (Vigna Taglianti 2005).

##### Notes

Macropterus. Eurytopic: fields, ruderals; from lowlands to mountains (Hůrka 1996).

#### Amara (Zezea) fulvipes

Audinet-Serville, 1821

234356E0-8CE5-5210-B486-0D32CA9743F9

##### Distribution

Southern Europe, south of Central Europe and Asia Minor (Hůrka 1996). It can be found in mainland Italy, Sardinia included (Vigna Taglianti 2005).

##### Notes

Lives in dry, unshaded habitats, steppe and fields; from lowlands to hills (Hůrka 1996).

#### Amara (Amara) similata

Gyllenhal, 1810

4BA92D21-AF64-5BE6-B39B-2F808076496A

##### Distribution

Transpalearctic species, ranging eastwards to Kamchatka (Hůrka 1996). It can be found in mainland Italy, Sicily and Sardinia (Vigna Taglianti 2005).

##### Notes

Macropterus. Lives in dry to moderately moist, unshaded habitats: fields, meadows, ruderals; from lowlands to mountains, mostly in hills (Hůrka 1996).

#### Anchomenus (Anchomenus) dorsalis

Pontoppidan, 1763

B1E6BFA3-39C9-559E-9453-F11554360E05

##### Distribution

West Palearctic species, ranging eastwards to Middle Asia (Hůrka 1996). It can be found in mainland Italy, Sicily and Sardinia (Vigna Taglianti 2005).

##### Notes

Macropterus. Lives in unshaded, dry to moderately moist habitats; fields, steppe, pastures, edges of small woods; from lowlands to mountains, often gregariously (Hůrka 1996).

#### Anisodactylus (Anisodactylus) binotatus

Fabricius, 1787

FB0DA50F-0BB8-5C3E-A8C3-BB3FC2D1A860

##### Distribution

West Palearctic species, ranging eastwards to Middle Asia (Hůrka 1996). It can be found in mainland Italy, Sicily and Sardinia (Vigna Taglianti 2005).

##### Notes

Macropterus. Eurytopic species: meadows, water margins with vegetation, fields and ruderals; from lowlands to mountains (Hůrka 1996).

#### Anisodactylus (Pseudanisodactylus) signatus

Panzer, 1796

6F510F64-B789-59AF-815F-704850E238E3

##### Distribution

Transpalearctic species (Hůrka 1996). It can be found in northern Italy (Vigna Taglianti 2005).

##### Notes

Macropterus. Lives in moderately dry to moist, unshaded habitats, mainly on sandy, loamy ground: grassy water edges, sand pits, fields, saline habitats; from lowlands to foothills (Hůrka 1996).

#### Badister (Badister) bullatus

Schrank, 1798

D8586A20-30F5-518F-A1D7-EC87AD301061

##### Distribution

Circumpolar and Paleartic species (Hůrka 1996). It can be found in mainland Italy and Sicily; doubtfully present in Sardinia (Vigna Taglianti 2005, Vigna Taglianti 2010).

##### Notes

Macropterus. Lives in dry to wet habitats, indifferent to shade: steppe, meadows, overgrown edges of water and swamps; from lowlands to mountain, mostly in hills (Hůrka 1996).

#### Bembidion (Bembidion) quadrimaculatum

Linnaeus, 1760

758147E6-3ADD-55ED-8C2E-E4D89D9053B2

##### Distribution

Holoarctic species (Hůrka 1996). It can be found in mainland Italy and Sicily (Vigna Taglianti 2005).

##### Notes

Macropterus. Lives in both drier and moist, unshaded or partly shaded habitats: fields, meadows, even far from water; from lowlands to mountains (Hůrka 1996).

#### Brachinus (Brachinus) elegans

Chaudoir, 1842

D2FFC41D-C4F4-5308-9F6B-EE4B97BA8E64

##### Distribution

Mediterranean species (Vigna Taglianti 2005). It can be found in mainland Italy, Sicily and Sardinia (Vigna Taglianti 2005).

##### Notes

It frequently lives in open formations of the western alpine valleys. It is present in damp meadows, but occasionally we can find it in biotopes characterised by relative xericity (Bisio 2011).

#### Brachinus (Brachynidius) sclopeta

Fabricius, 1792

14827DF5-B34C-52F5-943F-7E38ADB4281A

##### Distribution

European and Mediterranean species (Anderson 2005, Vigna Taglianti 2005). It can be found in mainland Italy, Sicily and Sardinia (Vigna Taglianti 2005).

##### Notes

Species widely widespread in the plains (Bisio 2011).

#### Calathus (Calathus) fuscipes

Goeze, 1777

9C3EEB30-8424-5E86-9BEB-7A3EDAE7D672

##### Distribution

West Palearctic species, introduced in North America (Hůrka 1996). It can be found in mainland Italy and Sicily; doubtfully present in Sardinia (Vigna Taglianti 2005, Vigna Taglianti 2010).

##### Notes

Brachypterus, rarely macropterous. Lives in unshaded, rather dry habitats: meadows, fields, balks and steppes; from lowlands to mountains. (Hůrka 1996).

#### Calathus (Neocalathus) melanocephalus

Linnaeus, 1758

1D7D0B6C-B8F1-5B4F-87A7-F146DA1E18EC

##### Distribution

Palearctic species, probably introduced into North America (Hůrka 1996). It can be found in mainland Italy (Vigna Taglianti 2005).

##### Notes

Brachypterus, less frequently macropterous. Lives mainly in unshaded or moderately-shaded habitats: fields, steppe; from lowlands to mountains (Hůrka 1996).

#### Calosoma (Campalita) auropunctatum

Herbst, 1784

F88049A4-5BA0-5F83-B494-2E0DE21E273F

##### Distribution

Europe, Asia Minor, Syria and Egypt (Hůrka 1996). In can be found in northern Italy (Vigna Taglianti 2005).

##### Notes

Lives in fields and steppe in lowlands (Hůrka 1996).

#### Carabus (Carabus) granulatus

Linnaeus, 1758

F3D4E99F-4F28-5972-A692-EE7C5CFDBFA4

##### Distribution

Transpalearctic species, distributed from Pyrenees and Great Britain as far east as Sakhalin and Japan. Introduced in North America (Hůrka 1996). It can be found in mainland Italy; it is widespread in the northen part (Vigna Taglianti 2005, Ruffo and Stoch 2005).

##### Notes

Brachypterous individuals dominate, but both macropterous and apterous specimens occurs. It is an hygrophilous, eurytopic species of both unshaded and shaded habitats; from lowlands to mountains (Hůrka 1996).

#### Chlaeniellus nitidulus

Schrank, 1781

29926319-BBB9-5D44-A99B-95F6D15EB14B

##### Distribution

European species (Hůrka 1996). It can be found in mainland Italy (Vigna Taglianti 2005).

##### Notes

Macropterous. Lives on unshaded, overgrown edges of waters, in meadows and clay pits; from lowlands to foothills, more often in hills (Hůrka 1996).

#### Clivina (Clivina) fossor

Linnaeus, 1758

C9313A9C-EBF1-5991-9563-EBAE7FEB7C3D

##### Distribution

Holarctic species, in Palearctic Region eastwards to Kamchatka and NE China (Hůrka 1996). It can be found in mainland Italy and Sicily; doubtfully present in Sardinia (Vigna Taglianti 2005).

##### Notes

Both brachypterous and macropterous. Lives on moist, unshaded or slightly shaded habitats: meadows and water shores; from lowlands to mountains (Hůrka 1996).

#### Diachromus germanus

Linnaeus, 1758

B5B535DB-C4AA-5A77-86A9-A1D4C39ABA5E

##### Distribution

West Palearctic species (Hůrka 1996). It can be found in mainland Italy, Sicily and Sardinia (Vigna Taglianti 2005).

##### Notes

Macropterous. Lives mostly in moist, unshaded habitats: water edges with vegetation, meadows along waters, saline habitats, overgrown bottoms of drained ponds; from lowlands to foothills (Hůrka 1996).

#### Dolichus halensis

Schaller, 1783

D7960EA2-E880-57C3-B44D-66D292E12E99

##### Distribution

Palearctic species, reaching southern Kuril Islands, Japan and South China (Hůrka 1996). It can be found in mainland Italy (Vigna Taglianti 2005).

##### Notes

Macropterous. Lives on dry to moderately dry, unshaded habitats: fields; from lowlands to hills (Hůrka 1996).

#### Egadroma marginatus

Dejean, 1829

E8986484-D1D2-5AD2-82CB-A7EA50640FD8

##### Distribution

It is a typically Mediterranean species, widespread in warm regions of the Western Palearctic (Chittaro and Marggi 2015). It can be found in mainland Italy, Sicily and Sardinia (Vigna Taglianti 2005).

##### Notes

It is generally described as riparian and swamp-dwelling species. It occurs near freshwater, particularly irrigation canals and swamps Chittaro and Marggi 2015).

#### Harpalus (Harpalus) affinis

Schrank, 1781

FC51AE3A-3AA0-537B-8B88-564B92D2CB83

##### Distribution

Transpalearctic species, introduced in North America (Hůrka 1996). It can be found in mainland Italy; doubtfully present in Sardinia (Vigna Taglianti 2005).

##### Notes

Macropterous. Lives in dry to moderately moist, unshaded habitats: fields, meadows and ruderals; from lowlands to mountains (Hůrka 1996).

#### Harpalus (Harpalus) distinguendus

Duftschmid, 1812

F7CEB853-D551-5132-B9F6-8EF997E20D08

##### Distribution

Transpalearctic species, ranging from the Azores and NW Africa to Far East (Hůrka 1996). It can be found in mainland Italy, Sicily and Sardinia (Vigna Taglianti 2005).

##### Notes

Macropterous. Live in dry to moderately moist, unshaded habitats: fields, steppes and ruderals; from lowlands to hills (Hůrka 1996).

#### Harpalus (Harpalus) oblitus

Dejean, 1829

557682E1-2C76-5DEC-B5D6-9C3DBB8DEE3F

##### Distribution

South-western Palearctic Region, from north-western Africa and Iberian Peninsula to south-western Siberia and south-eastern Kazakhstan (Hůrka 1996). It can be found in mainland Italy, Sicily and Sardinia (Vigna Taglianti 2010, Vigna Taglianti 2005).

##### Notes

Macropterous. Lives in moderately dry, unshaded habitats: vineyards, balks and steppes; it can be found in lowlands (Hůrka 1996).

#### Harpalus (Harpalus) serripes

Quensel in Schonherr, 1806

3C2C3AF3-F0EC-538C-A679-554212ED8F08

##### Distribution

West Palearctic species, reaching Caucasus and Middle Asia (Hůrka 1996). It can be found in mainland Italy and Sicily; doubtfully present in Sardinia (Vigna Taglianti 2005).

##### Notes

Macropterous. Lives in very dry to moderately dry, unshaded habitats: steppe, fields and quarries; from lowlands to foothills (Hůrka 1996).

#### Limodromus (Platynus) assimilis

Paykull, 1790

2A089CD3-854B-5310-AE68-53D240964F5F

##### Distribution

Transpalearctic species, distributed eastwards to Sakhalin and Japan (Hůrka 1996). It can be found in mainland Italy (Vigna Taglianti 2005).

##### Notes

Macropterous. Lives indifferent to moisture or very moist habitats, to entirely shaded: forests, parks and shaded water edges; from lowlands to mountains, often in hills (Hůrka 1996).

#### Limodromus (Platynus) krynickii

Sperk, 1835

4F3B6745-8BA6-5551-9C25-3E5C3C0A19FC

##### Distribution

West Palearctic species (Hůrka 1996). It can be found in mainland Italy (Vigna Taglianti 2005).

##### Notes

Macropterous. It is a hygrophilous species of shaded borders of waters with rich vegetation in floodplain forests; it can be found in lowlands (Hůrka 1996).

#### Metallina (Metallina) properans

Stephens, 1828

7A51A8A2-05B4-59E8-A940-C42217541486

##### Distribution

Palearctic species introduced in North America (Hůrka 1996). It can be found in mainland Italy (Vigna Taglianti 2005).

##### Notes

Macropterous, rarely brachypterous. It lives in unshaded, drier or moist habitats: meadows, grassy, loamy edges of water; from lowlands to foothills (Hůrka 1996).

#### Microlestes minutulus

Goeze, 1777

3C6B0707-AB24-5BCA-A305-012537885826

##### Distribution

Palearctic species introduced into North America and India (Hůrka 1996). It can be found in mainland Italy; doubtfully present in Sicily and Sardinia (Vigna Taglianti 2005).

##### Notes

Macropterous, rarely brachypterous. Lives indifferent to shade: steppe and forests; from lowlands to mountains, often in hills (Hůrka 1996).

#### Panagaeus (Panagaeus) cruxmajor

Linnaeus, 1758

BDC1E8C0-80FC-544E-9BB2-DC6E83FD2B1D

##### Distribution

Palearctic species (Hůrka 1996). It can be found in mainland Italy, Sicily and Sardinia (Vigna Taglianti 2005).

##### Notes

Macropterous. Lives in moist, unshaded habitats: meadows near waters and grassy water edges; from lowlands to hills (Hůrka 1996).

#### Paranchus albipes

Fabricius, 1796

59B8FF1C-FDFD-5B6F-B8A5-E61D0DA43E16

##### Distribution

Europe, North Africa and Asia Minor, introduced into North America (Hůrka 1996). It can be found in mainland Italy, Sicily and Sardinia (Vigna Taglianti 2005).

##### Notes

Macropterous. Often lives on gravelly habitats, gravelly sandy, gravelly loamy to stony water edges, largely tolerating pollution; from lowlands to mountains. Indifferent to shade (Hůrka 1996).

#### Parophonus (Ophonomimus) hirsutulus

Dejean, 1829

2D436360-467C-5B80-BDA3-FC792C72FAEC

##### Distribution

Turanic-Mediterranean (Pilon et al. 2013). It can be found in mainland Italy, Sicily and Sardinia (Vigna Taglianti 2005).

##### Notes

Macropterous. It is a hygrophilous species, lives in open habitats (Pilon et al. 2013).

#### Parophonus (Parophonus) maculicornis

Duftschmid, 1812

DC8A171C-3E38-50C5-AB13-A8F0D1E18D18

##### Distribution

South-western part of the Paleartic Region, eastwards to Caucasus and Syria (Hůrka 1996). It can be found in mainland Italy, Sicily and Sardinia (Vigna Taglianti 2005).

##### Notes

Macropterous. Lives indifferent in the shade: steppe, vineyards and floodplain groves; it can be found in lowlands (Hůrka 1996).

#### Poecilus (Poecilus) cupreus

Linnaeus, 1758

9EC31B50-4844-515D-9782-529293B02508

##### Distribution

West Palearctic species, reaching Central Siberia and Middle Asia (Hůrka 1996). It is widespread throughout Italy, Sicily and Sardinia included (Ruffo and Stoch 2005, Vigna Taglianti 2005).

##### Notes

Macropterous. Lives in unshaded habitats: fields, steppes and water edges; from lowlands to mountains (Hůrka 1996).

#### Poecilus (Poecilus) versicolor

Sturm, 1824

E4B9B717-0BFB-538E-B462-07205C753A25

##### Distribution

Palearctic species (Hůrka 1996). It can be found in mainland Italy; it is widespread in northern parts (Ruffo and Stoch 2005, Vigna Taglianti 2005).

##### Notes

Macropterous. Lives in unshaded habitats: meadows, pastures, fields, water edges with vegetation and forest clearings; from lowlands to mountains, mostly in hills (Hůrka 1996).

#### Pseudoophonus (Pseudoophonus) griseus

Panzer, 1796

EB0347E6-3B1A-585E-8EAD-8E5387F98B07

##### Distribution

Transpalearctic species (Hůrka 1996). It can be found in mainland Italy, Sicily and Sardinia (Vigna Taglianti 2005).

##### Notes

Macropterous. Lives in dry to indifferent, unshaded habitats: fields, meadows, ruderals and often on sandy soil; from lowlands to mountains (Hůrka 1996).

#### Pseudoophonus (Pseudoophonus) rufipes

De Geer, 1774

0FBEE799-341A-5515-8B1A-DC5741FF690F

##### Distribution

Palearctic species introduced into North America (Hůrka 1996). It is widespread throughout Italy, Sicily and Sardinia included (Vigna Taglianti 2005).

##### Notes

Macropterous. Lives in dry to moderately moist, preferably unshaded habitats: fields, meadows, ruderals and forest edges; from lowlands to mountains (Hůrka 1996).

#### Pterostichus (Melanius) aterrimus

Herbst, 1784

5868B544-B36C-5580-9072-40C52071DBB9

##### Distribution

West Palearctic species (Hůrka 1996). It can be found in mainland Italy (Vigna Taglianti 2005).

##### Notes

Macropterous. Lives on borders of swamps amongst soaking wet vegetation; it can be found in lowlands (Hůrka 1996).

#### Pterostichus (Morphnosoma) melanarius

Illiger, 1798

0B920836-F222-5AD0-894B-1FDF23245985

##### Distribution

Eurosiberian species, introduced ino North America (Hůrka 1996). It can be found in mainland Italy (Vigna Taglianti 2005).

##### Notes

Brachypterous, rarely macropterous. It is a very eurytopic species of fields, meadows, gardens, as well as forests; from lowlands to mountains (Hůrka 1996).

#### Pterostichus (Platysma) niger

Schaller, 1783

A859DD82-34B1-5851-BBF6-1EB88142310C

##### Distribution

Palearctic species (Hůrka 1996). It can be found in mainland Italy and Sardinia (Vigna Taglianti 2005).

##### Notes

Macropterous. Lives in moist habitats, indifferent to shade: meadows, forests, water margins with vegetation; from lowlands to mountains, frequently in hills (Hůrka 1996).

#### Pterostichus (Phonias) strenuus

Panzer, 1796

F2D5F44E-1B9D-59F4-AA02-505CD34F2D9D

##### Distribution

Eurosiberian species (Hůrka 1996). It can be found in mainland Italy (Vigna Taglianti 2005).

##### Notes

Brachypterous, more frequently macropterous. Prefers moist habitats, indifferent to shade: floodplain forests, meadows near water, margins of waters with vegetation and forest clearings; from lowlands to mountains (Hůrka 1996).

#### Pterostichus (Argutor) vernalis

Panzer, 1796

902DF406-B9E4-5A10-842B-9D73A1B58FA5

##### Distribution

Palearctic species (Hůrka 1996). It can be found in mainland Italy (Vigna Taglianti 2005).

##### Notes

Macropterous. Lives in moist to wet habitats, indifferent to shade: grassy water shores, moist meadows, floodplain forests and gardens; from lowlands to mountains (Hůrka 1996).

#### Sphaerotachys hoemorrhoidalis

Ponza, 1805

5553F5C8-630F-5E7A-8FCB-760C7BFAB811

##### Distribution

Mediterranean and Caucasian species (Hůrka 1996). It can be found in mainland Italy, Sicily and Sardinia (Vigna Taglianti 2005).

##### Notes

Macropterous. Lives on marshy borders of irrigation canal; it can be found in lowlands (Hůrka 1996).

#### Stenolophus (Stenolophus) teutonus

Schrank, 1781

2D87C131-99BD-5A80-A913-2053835A11CC

##### Distribution

West Palearctic species (Hůrka 1996). It can be found in mainland Italy, Sicily and Sardinia (Vigna Taglianti 2005).

##### Notes

Macropterous. Lives on unshaded, overgrown edges of waters; from lowlands to foothills (Hůrka 1996).

#### Lucanidae

D8704C36-EE14-55ED-83CF-00C203464AE2

#### Dorcus parallelipipedus

Linnaeus, 1785

B63E988B-52BD-5104-918A-DFDB594BE24C

##### Ecological interactions

###### Conservation status

Least Concern for Italian (Audisio et al. 2014) and European assessments (Clix et al. 2018).

##### Distribution

West Palearctic species (Bartolozzi and Sprecher-Uebersax 2006).

##### Notes

It has a larval biology linked to the presence of rotting wood in deciduous and sometimes coniferous forests (Boucher 2014, Franciscolo 1997).

#### Staphilinidae

6C6D0725-F739-5BBE-934F-492E89124C49

#### Aleochara (Coprochara) bipustulata

Linnaeus, 1760

797DA1C7-E55E-5FE9-ACAE-D3ED49103ADD

##### Distribution

Palearctic species (Maus 1998). In Italy, it is widespread (Ruffo and Stoch 2005).

##### Notes

Lives in open habitats. In particular, adults can be found in rotting *Brassica* and other vegetation and it is associated with carrion and dung (Andreassen 2013).

#### Anotylus rugosus

Fabricius, 1775

76CB8FFF-D088-5E3F-BD44-8699EA73936D

##### Distribution

Palearctic species (Majka and Klimaszewski 2012). Widespread in Italy, but absent in Sicily (Ruffo and Stoch 2005).

##### Notes

Frequently found on dung, carrion and other decomposing organic matter (Majka and Klimaszewski 2012).

#### Arpedium quadrum

Gravenhorst, 1806

AFC83993-BC3B-57F2-8817-0E3ABD1B5498

##### Distribution

Europe and Russian species (ITIS Species 2000 2020). The presence of this species is reported only for northern Italy including: Friuli Venezia Giulia, Veneto, Trentino-Alto Adige, Lombardia, Val d'Aosta, Piemonte, Liguria and Emilia-Romagna (Ruffo and Stoch 2005).

##### Notes

Lives on banks, riverbanks, marshy areas (*Phragmitetum*, *Caricetum*), wetlands, damaged marshy areas, rural settlements, sandy small areas and wetlands near source (Zanetti et al. 2016).

#### Astrapaeus ulmi

Rossi, 1790

9763E213-BC8F-5DF7-AEAB-C7A15BA0C89D

##### Distribution

Europe (except in the northern part) and it reaches western Turkey (Pietrykowska-Tudruj et al. 2014). Its occurrence is patchy and it is generally considered a rare species (Assing and Schülke 2012). In Italy, the species is present along the peninsula, in Sicily and in Sardinia (Ruffo and Stoch 2005).

##### Notes

As a thermophilus species, inhabits xerothermic habitats with moderately moist soil. Many authors collected the adults in open grassy sites with some layers of humus, under heaps of rotting plants or stones and often in riverside areas covered with low vegetation (Pietrykowska-Tudruj et al. 2014).

#### Atheta (Atheta) aeneicollis

Sharp, 1869

2DC08EBA-79BB-5C65-A70C-13E0DB76C4F8

##### Distribution

West Palearctic species (Sushko 2016): Europe, Canary Islands, western North Africa, Cyprus, Israel and Syria (Zanetti et al. 2016). In Italy, the species is widespread (Ruffo and Stoch 2005).

##### Notes

Lives on banks, forests (floodplain, hill and mountains), reforestation areas (*Picea, Pinus*), pebbly shores, lakeshores, pastures, wetlands, clearings, meadows, margin of pastures with trees, gorges, hill bushes, historical gardens, vineyards, lakeshore with *Phragmitetum*, rural settlements, riverbanks, floodplains, uncultivated areas, orchards, marshy areas (*Phragmitetum, Caricetum*), vegetable crops, subalpine bushes (*Alnus*) and corn crops (Zanetti et al. 2016).

#### Atheta fungii

Gravenhorst, 1806

1A6C24A5-46F7-52C0-B6FA-753921EF413B

##### Distribution

Under the name *Atheta fungii*, considered a widespread Palaearctic species, a very difficult complex of taxa is included (Zanetti et al. 2016, Sushko 2016). In Italy, the species is widespread (Ruffo and Stoch 2005).

##### Notes

Lives on banks, pastures, forests (floodplain, hill and mountains), reforestation areas (*Picea, Pinus*), gardens, wetlands, subalpine bushes (*Pinus mugo*), peat bogs, meadow, parks, margin of pastures with trees, subalpine grasslands, waterfalls, historical gardens, uncultivated areas, marshy areas (*Phragmitetum*, *Caricetum*), riverbanks, floodplains, orchards, vegetable crops, rural settlements and rows of trees (*Quercus*) (Zanetti et al. 2016).

#### Atheta (Dimetrotina) laticollis

Stephens, 1832

A8141A04-00F1-5C4F-90E8-1AA0549AFFC4

##### Distribution

European species (ITIS Species 2000 2020). In Italy, the species is widespread (Ruffo and Stoch 2005).

##### Notes

Lives in clearing, hill forests, lakeshores, pastures, wetlands, subalpine bushes (*Pinus mugo*), reforestation areas (*Pinus nigra*), mountain forests (*Fagus*), banks, parks, damaged hill forests, rural settlements, riverbanks, gardens, marshy areas (*Phragmitetum*, *Caricetum*) and uncultivated areas (Zanetti et al. 2016).

#### Atheta (Atheta) triangulum

Kraatz, 1856

94620AC0-37A3-5563-8FE8-0FC4E1EAAA36

##### Distribution

Palearctic species (Lupi et al. 2006). In Italy, the species is widespread (Ruffo and Stoch 2005).

##### Notes

It is a saprophilous species widespread from the plains to middle altitude elevations (1000 m). It is a very common predator and it is found mostly in decaying vegetable matter (Lupi et al. 2006).

#### Carpelimus (Taenosoma) corticinus

Gravenhorst, 1806

ED6C4861-DF11-5B8A-99D8-B3788099A51D

##### Distribution

Cosmopolitan species (ITIS Species 2000 2020). In Italy, the species is widespread (Ruffo and Stoch 2005).

##### Notes

Lives on banks, pebbly shores, cirques, coppiced mixed forests, wetlands, hill forests, parks, mountain forests (*Fagus*), pastures, *Phragmitetum*, riverbanks, floodplain forests, marshy areas (*Phragmitetum*, *Caricetum*), damaged marshy areas, gardens, uncultivated areas, vegetable crops and wetlands near source (Zanetti et al. 2016).

#### Cordalia obscura

Gravenhorst, 1802

4F5ACF91-C27C-5072-BFC6-9D189C3DBC70

##### Distribution

Europe, north Asia (excluding China), Caucaso and North America (Pace 2005, Majka and Klimaszewski 2012). In Italy, the species is widespread (Ruffo and Stoch 2005).

##### Notes

Common in decaying plant material and also found on carrion and dung. It is found in the nest of several birds species (Majka and Klimaszewski 2012).

#### Dinaraea angustula

Gyllenhal, 1810

74C983CC-D14B-5C7B-977C-F66DBF7B4A2D

##### Ecological interactions

###### Conservation status

Least Concern for Italian assessment (Audisio et al. 2014).

##### Distribution

Europe, Turkey and North America (ITIS Species 2000 2020). The species is present only in the Italian pensinsula (absent in the Islands) (Ruffo and Stoch 2005).

##### Notes

Lives in parks, banks, wetlands, floodplains, orchards, marshy areas (*Phragmitetum*, *Caricetum*), damaged marshy areas, vegetable crops, rural settlements, permanent meadows, wetlands near source and vineyards (Zanetti et al. 2016).

#### Drusilla (Drusilla) canaliculata

Fabricius, 1787

751139B6-7D43-5AA2-B1FC-4E3AC9FA07DF

##### Distribution

Palearctic species (Majka and Klimaszewski 2012): Central-southern Europe, Russia (European, Siberia, Far East), Ukraine, Armenia, Georgia, Turkey, Azerbaijan, Israel, Iran, Kazakhstan, Kyrgyzstan, Korea, Japan and Nepal; introduced into Canada (ITIS Species 2000 2020, Pace 2005). In Italy, the species is currently known only for the peninsula (Ruffo and Stoch 2005).

##### Notes

Found in open areas under vegetation, stone, mosses and decomposing materials. A very eurytopic species adapted to a wide range of ground conditions: it can be found on dry, heathy and sandy soils; on damp loam and in humic soils; in wet soil and *Sphagnum*. Often found in proximity of ants (Majka and Klimaszewski 2012).

#### Euryalea murina

Erichson, 1839

B9AE841B-BB07-56F2-B632-08BE154435D9

##### Distribution

European species (ITIS Species 2000 2020). In Italy, it is widespread (Ruffo and Stoch 2005).

##### Notes

Lives in banks of stream, pastures, hill forests, parks, coppiced mixed forests, uncultivated areas, marshy areas (*Phragmitetum*, *Caricetum*), permanent meadows, wetlands near source (Zanetti et al. 2016).

#### Falagria sulcatula

Gravenhorst, 1806

8024D208-92AF-5F33-9735-8408F748E589

##### Distribution

Holoarctic species (ITIS Species 2000 2020). Widespread in Italy, but absent in Sicily (Ruffo and Stoch 2005).

##### Notes

Lives in banks, wetlands, orchards, vegetable crops, marshy areas (*Phragmitetum*, *Caricetum*) and permanent meadows (Zanetti et al. 2016).

#### Falagrioma thoracica

Stephens, 1832

060D8392-DE70-5FF8-971F-D0B0780AB2B3

##### Distribution

Palearctic species (ITIS Species 2000 2020). In Italy, it is widespread (Ruffo and Stoch 2005).

##### Notes

Lives in banks, hill forests (*Quercus ilex*), vineyards, mountain forests (*Abies*), historical gardens, mixed forests, floodplain forests, marshy areas (*Phragmitetum*, *Caricetum*), wetlands, parks, permanent meadows and wetlands near source (Zanetti et al. 2016).

#### Ochthephilum brevipenne

Mulsant & Rey, 1861

D1EE5038-191A-5AFE-8A31-896602184F88

##### Distribution

Mediterranean species, from south-western France and north-western Africa to Greece and Ukraine (including Italy and Switzerland) (ITIS Species 2000 2020, Zanetti et al. 2016).

##### Notes

Lives in banks, coppiced mixed forests, marshy areas (*Phragmitetum*, *Caricetum*), permanent meadows, lakeshore with *Phragmitetum*, orchards and wetlands (Zanetti et al. 2016).

#### Ocypus (Matidus) brunnipes

Fabricius, 1781

24F5F913-6703-5A61-894F-6B5770C31E47

##### Distribution

Palearctic species (ITIS Species 2000 2020). The species is present only in the Italian pensinsula (absents in the Islands) (Ruffo and Stoch 2005).

##### Notes

Lives amongst the stones, mosses and plant remains (Fernàndez and Masò 2010).

#### Ocypus (Ocypus) olens

O.F.Müller, 1764

263A237C-83D2-5C0A-9551-57D440578365

##### Distribution

Euro-Mediterranean species (ITIS Species 2000 2020). In Italy, it is widespread (Ruffo and Stoch 2005).

##### Notes

Lives on lakeshore, mountain forests (*Abies*), vineyards, dry meadows, hill forests (*Carpinus*), margin of pastures with trees, coppiced mixed forests, mixed forests, hill bushes, wetlands, floodplain forests, floodplains, banks, riverbanks, marshy areas (*Phragmitetum*, *Caricetum*), orchards, sandy small areas, vegetable crops, wetlands near source and cellars (Zanetti et al. 2016).

#### Omalium caesum

Gravenhorst, 1806

0A1FF58C-422F-532A-81EF-F88300FB1747

##### Distribution

Holoarctic species (Sushko 2016). The species is widespread in the Italian pensinsula, while absent in the Islands (Ruffo and Stoch 2005, Zanetti et al. 2016).

##### Notes

It occurs in open bogs with *Carex* sp. and *Eriophorum vaginatum* (Sushko 2016).

#### Omalium oxyacanthae

Gravenhorst, 1806

596F16A8-5B6F-5995-85AC-AA4587AD2589

##### Distribution

Holoarctic species (ITIS Species 2000 2020). In Italy, it is widespread (Ruffo and Stoch 2005).

##### Notes

Lives in coppiced mixed forests, wetlands, floodplain, marshy areas (*Phragmitetum*, *Caricetum*), sandy small areas, uncultivated areas and wetlands near source (Zanetti et al. 2016).

#### Oxypoda (Oxypoda) opaca

Gravenhorst, 1802

DF4E3333-85CF-598C-9F4E-03655C1787C4

##### Distribution

Palearctic species (ITIS Species 2000 2020). In Italy, it is widespread (Ruffo and Stoch 2005).

##### Notes

Lives in banks, reforestation areas (*Pinus nigra*), forests (hill and mountain), meadows, wetlands, disused quarries, pastures, parks, marshy areas (*Phragmitetum*, *Caricetum*), riverbanks, orchards, gardens, uncultivated areas, corn crops and wetlands near source (Zanetti et al. 2016).

#### Paederus (Heteropaederus) fuscipes

Curtis, 1826

3DE3997F-1AB4-5648-A87C-41271B9912F5

##### Distribution

Africa, Asian and European species (Smetana 2004). In Italy, it is widespread (Ruffo and Stoch 2005).

##### Notes

Lives in banks, coppiced mixed forests, subalpine bushes (Rhododendron), peat bogs, parks, cultivated riverbanks, permanent meadows, lakeshore with *Phragmitetum*, marshy areas (*Phragmitetum*, *Caricetum*), wetlands, floodplain forests, damaged marshy areas, rural settlements, uncultivated areas, riverbanks, wetlands near source and vineyards (Zanetti et al. 2016).

#### Philonthus (Philonthus) carbonarius

Gravenhorst, 1802

4FD09814-7A4E-50FB-957F-17E88EBBDB69

##### Distribution

Siberian-European species (Sushko 2016). In Italy, it is widespread (Ruffo and Stoch 2005).

##### Notes

Widespread, but not abundant in open bogs (Sushko 2016).

#### Philonthus (Philonthus) cognatus

Stephens, 1832

CB690A70-9785-5060-B2E4-BA56DCF4EAD8

##### Distribution

Holoarctic species (Sushko 2016). In Italy, it is widespread (Ruffo and Stoch 2005).

##### Notes

It is a widespread species that lives in all bog habitats (Sushko 2016).

#### Philonthus (Philonthus) succicola

C.G.Thomson, 1860

D7FCA175-7EC4-5337-80F8-CB8C6E3771C6

##### Distribution

Paleartic species. Widespread in Italy but absent in Sardinia (Ruffo and Stoch 2005).

##### Notes

Lives on putrefying matter and dung (Schmidt 2006).

#### Platystethus (Craetopycrus) burlei

Brisout de Barneville, 1862

E5F5D223-9119-58DE-B0B8-EEBE20A45199

##### Distribution

European species (ITIS Species 2000 2020). Widespread in Italy, but absent in Sardinia (Ruffo and Stoch 2005).

#### Platystethus (Craetopycrus) nitens

C. Sahlberg, 1832

ABF0C86F-C929-5C8E-8C00-A5A46CE4AB9E

##### Distribution

Palearctic species (ITIS Species 2000 2020). In Italy, it is widespread (Ruffo and Stoch 2005).

##### Notes

Lives on banks, pebbly shores, gorge with rocks, forests (hill and mountain), reforestation areas (*Picea, Pinus*), fountains, pastures, lakeshores, wetlands, cirques, subalpine bushes (*Rhododendron*, *Dryas*, *Pinus mugo*), peat bogs, screes, parks, coppiced mixed forests, dry meadows, marshy areas (*Phragmitetum*, *Caricetum*), rural settlements, riverbanks, vegetable crops, uncultivated areas, corn crops and wetlands near source (Zanetti et al. 2016).

#### Proteinus ovalis

Stephens, 1834

DDD9E965-C302-55B4-A035-64F7C296E6ED

##### Distribution

European species (ITIS Species 2000 2020). In Italy, it is widespread (Ruffo and Stoch 2005).

##### Notes

Lives forests (floodplain, mountain and hill)) , banks, screes, reforestation areas (*Picea, Pinus*), pastures, banks of stream, parks, subalpine bushes (*Pinus mugo*, *Alnus*), disused quarries, dry meadows, margin of pastures with trees, subalpine grasslands, tumbled limestones, row of trees (*Quercus*), caves, meadows, hill bushes, historical gardens, vineyards, wetlands, lakeshore with *Phragmitetum*, floodplains, riverbanks, marshy areas (*Phragmitetum*, *Caricetum*), damaged marshy areas, orchards, sandy small areas, vegetable crops, permanent meadows and wetlands near source (Zanetti et al. 2016).

#### Quedius cruentus

Olivier, 1795

7B203BA1-5AAC-547E-A536-F0E67F515883

##### Ecological interactions

###### Conservation status

Least Concern for Italian assessment (Audisio et al. 2014).

##### Distribution

Palearctic species (Zanetti 2007). In Italy, it is present along the peninsula and in Sicily (Ruffo and Stoch 2005).

##### Notes

Lives in the rotting wood of several broad-leaved species (Zanetti 2007).

#### Quedius (Raphirus) nitipennis

Stephens, 1833

695FF98D-2524-57F2-9931-D6AF99305F57

##### Distribution

Palearctic species (ITIS Species 2000 2020). Widespread in Italy but absent in Sardinia (Ruffo and Stoch 2005).

##### Notes

Lives in pastures, peat bogs, mountain forests (*Fagus*), meadows, banks, wetlands, uncultivated areas, marshy areas (*Phragmitetum*, *Caricetum*) and rural settlements (Zanetti et al. 2016).

#### Rugilus (Rugilus) orbiculatus

Paykull, 1789

0031C1C1-B049-53E7-B1FB-FF7DC49371EC

##### Distribution

Holoarctic species (ITIS Species 2000 2020). Widespread in Italy but absent in Sardinia where it is replaced by the subspecies *Rugilus orbiculatus sardous* (Ruffo and Stoch 2005).

##### Notes

Lives in olive groves, banks, clearings, coppiced mixed forests, rural settlements, vineyards, parks, wetlands, riverbanks, marshy areas (*Phragmitetum*, *Caricetum*), sandy small areas, uncultivated areas, wetlands and marshy areas near source (Zanetti et al. 2016).

#### Tachinus (Tachinus) corticinus

Gravenhorst, 1802

361BE466-7DB9-51B6-8D4A-E5B1EAD777F4

##### Distribution

Europe, Russia, south to Turkey and the Caucasus, east to Japan (Majka and Klimaszewski 2012). The presence of this species is reported only for northern Italy including: Friuli Venezia Giulia, Veneto, Trentino-Alto Adige, Lombardia, Val d'Aosta, Piemonte, Liguria and Emilia-Romagna (Ruffo and Stoch 2005).

##### Notes

Lives in both lowlands and mountain areas, mainly in moist mixed and deciduous forests. It can be found under fallen leaves, in mosses, in compost and rotting hay and straw, and in mountains under stones in moist places (Majka and Klimaszewski 2012).

#### Xantholinus (Xantholinus) linearis

Olivier, 1795

A5C7D7B5-4B55-5F02-9786-527CF6DB348D

##### Distribution

Palearctic species (Majka and Klimaszewski 2012). Widespread in Italy, but absent in Sardinia (Ruffo and Stoch 2005).

##### Notes

Found in many kinds of decaying organic matter such as animal dung, compost piles and decaying vegetation; also in leaf litter and debris and amongst low vegetation in moist habitats; often around gardens and farmhouses (Majka and Klimaszewski 2012).

#### Xantholinus (Xantholinus) longiventris

Heer, 1839

47D36F59-2767-5450-87C6-98C7D083F80D

##### Distribution

Holoarctic species (ITIS Species 2000 2020). Widespread in Italy, but absent in Sardinia (Ruffo and Stoch 2005).

##### Notes

Lives on banks, clearings, meadows, dry meadows, coppiced mixed forests, lakeshore with *Phragmitetum*, wetlands, riverbanks, floodplains, floodplain forests, marshy areas (*Phragmitetum*, *Caricetum*), orchards, parks, vineyards, damaged marshy areas, vegetable crops, permanent meadows, rural backyards, rural settlements and uncultivated areas (Zanetti et al. 2016).

### Lepidoptera

#### Carabidae

DB6F6EF7-6E62-52CC-A719-1058A4C4B5E8

#### Loxostege sticticalis

Linnaeus, 1761

10F837F9-D9CF-56DE-85C2-F9B959E9700F

##### Distribution

Holoarctic species (Fabiano 2017).

##### Notes

It is a migratory pest that causes serious economic damage every year. Seems to be polyphagous in its larval stage, but it has been reported to have obvious hostplant selection for many crops (sugar beet, potato and soybean) and pastures (Wei et al. 2017).

#### Pyrausta despicata

Scopoli, 1763

7CE6C887-9BC3-5422-A414-6E855C838D0C

##### Distribution

Holoarctic species (Fabiano 2017).

##### Notes

Euryecious and heliophilous species. The larvae feed on various species of *Plantago* spp., *Antennaria* spp. and *Salvia* spp. (Fabiano 2017).

#### Geometrida

D1D06218-6232-5430-A118-C92E8F3C6D36

#### Idaea deversaria

Herrich-Schäffer, 1847

CB89B422-F336-5A01-B90E-812D0B07CE6A

##### Distribution

European and Central Asian species. It can be found in mainland Italy, Sardinia and Sicily (Flamigni and Bastia 1998).

##### Notes

Common from the foothills up to about 1400 m. In the hills, it flies from early June to the first ten days of July, in the mountains throughout the month of July (Flamigni and Bastia 1998).

#### Timandra comae

Schmidt, 1931

074938EC-6338-508B-80C8-C600487D3016

##### Distribution

From North Africa across Europe to East Asia (Wagner 2020).

##### Notes

Inhabits many habitats in which the larval host plants (*Rumex* spp.) occurs, such as woodlands clearings and edges, extensively-managed meadows and pastures or shores and wetlands. Has 2-3 (in the south also 4) generations from May to September. The caterpillar overwinters. It is observed quite frequently on leaves and fruit stands (Wagner 2020).

#### Lycaenidae

27CBD268-4060-511E-B10F-8F68F9FE2667

#### Lycaena dispar

Haworth, 1802

6906B40A-8899-57B6-84C2-83D12AEB6C73

##### Ecological interactions

###### Conservation status

Least Concern for Italian (Balletto et al. 2015) and European assessments (Van Swaay et al. 2010); Near-Threatened for Global assessment (Gimenez 1996).

##### Distribution

It is distributed in Europe and north Turkey (Tolman and Lewington 2008). It can be found in northern and central Italy. It is present in the Po Valley and in the wetlands of northern Tuscany. The population of Lazio (Paludi Pontine) became extinct during the first half of the 1900s (Villa et al. 2009, IUCN, 2020 2020).

##### Notes

Hygrophilous species, it lives in humid meadows from the plain up to 500m a.s.l. Trivoltine species, with generations in April-May, June-July, August-September (Paolucci 2013, Villa et al. 2009).

#### Lycaena phlaeas

Linnaeus, 1761

4E66A2E6-75AB-5B25-8101-AA699A079929

##### Ecological interactions

###### Conservation status

Least Concern for Italian (Balletto et al. 2015), Mediterranean (Numa et al. 2016) and European assessments (Van Swaay et al. 2010).

##### Distribution

It is distributed in Canary Islands, North and east-central Africa, Europe, temperate Asia, Japan and northeast America (Tolman and Lewington 2008). It can be found in mainland Italy, Sardinia and Sicily (IUCN, 2020 2020, Villa et al. 2009).

##### Notes

Eurycora species widespread and locally common, it frequents flowery meadows, open hedges, grasslands with scattered patches, pastures, moors and grassy banks, from the plain up to 2000 m a.s.l. Trivoltine species with generations in April-May, June-July, August-September (Paolucci 2013, Villa et al. 2009).

#### Noctuidae

DC6AE818-7915-5F22-B8C4-7152FA0648BA

#### Autographa gamma

Linnaeus, 1758

7599FA02-B36F-5C1D-8623-2A099334B557

##### Distribution

Holarctic species (Plepys et al. 2002).

##### Notes

It is one of the largest migratory moths in the world. It is common and often abundant. Polyvoltine, flies almost all year round both at night and during the day. It frequents a great variety of environments because its larvae feed on many different herbaceous species (Sterry and Mackay 2005).

#### Rivula sericealis

Scopoli, 1763

09EE7C7D-DA69-540C-8CCE-0773FEDDBD2E

##### Distribution

European species (Shaw 2012).

##### Notes

It is a small, partly plurivoltine noctuid moth. It is generally occurring in a wide variety of grassy sites, perhaps most commonly in somewhat sheltered situations. The larvae feed on various grasses and it is also recorded from sedges. The adults are long-lived and there is then a succession of at least partial additional generations, such that adults are on flights more or less continuously from late May well into September, with summer generation larvae in various stages of growth from June until the third instar larvae start to enter diapause in the autumn (Shaw 2012).

#### Nymphalidae

B50CC094-E940-57BF-A943-B36F625E75DD

#### Aglais io

Linnaeus, 1758

6530EECC-B822-584B-A959-4682BA79A09D

##### Ecological interactions

###### Conservation status

Least Concern for Italian (Balletto et al. 2015), Mediterranean (Numa et al. 2016) and European assessments (Van Swaay et al. 2010).

##### Distribution

It is distributed in most of Europe south to northern half of Iberian peninsula, Sierra Nevada, north and central Greece, European Turkey and Mediterranean Islands. (Tolman and Lewington 2008). It can be found in mainland Italy, Sardinia and Sicily (Villa et al. 2009).

##### Notes

Eurycora species, it lives in open and sunny places, in the woods, wooded banks, humid meadows, uncultivated fields and disturbed ground, rocky gullies sheltered with bushes and small trees at the upper limit of the altitude. It is spread from the plain at 2500 m a.s.l. Univoltine species flies in June-July (Paolucci 2013, Villa et al. 2009).

#### Vanessa cardui

Linnaeus, 1758

AF13FBD0-AEE4-5DCD-8B2E-1AC427779AB4

##### Ecological interactions

###### Conservation status

Least Concern for Italian (Balletto et al. 2015), Mediterranean (Numa et al. 2016) and European assessments (Van Swaay et al. 2010).

##### Distribution

It is distributed in Europe and North Africa (Sterry and Mackay 2005). It can be found in mainland Italy, Sardinia and Sicily (Villa et al. 2009).

##### Notes

Migratory species widely distributed in many different environments, from the plains to 2500 m a.s.l. Migratory individuals arriving in Italy have two generations in June-July and September-October (Paolucci 2013, Villa et al. 2009).

#### Pieridae

431CE890-D87C-5C08-8341-F4E72584D77C

#### Colias alfacariensis

Ribbe, 1905

91F11DEC-153E-5C76-A6DF-D7A13CFA96FC

##### Ecological interactions

###### Conservation status

Least Concern for Italian (Balletto et al. 2015), Mediterranean (Numa et al. 2016) and European assessments (Van Swaay et al. 2010).

##### Distribution

It is distributed in south and central Europe and Turkey (Tolman and Lewington 2008). It can be found in mainland Italy, Sardinia and Sicily (Villa et al. 2009).

##### Notes

Thermophilic migratory species, it is found in arid grasslands, stony grasslands and rocky slopes from the plain at 1900 m a.s.l. Trivoltine species with generations in April-May, June-July and August-September (Paolucci 2013, Villa et al. 2009).

#### Colias crocea

Fourcroy, 1785

04CE3A6D-9D25-5076-8773-DF3C01A4F72C

##### Ecological interactions

###### Conservation status

Least Concern for Italian (Balletto et al. 2015), Mediterranean (Numa et al. 2016) and European assessments (Van Swaay et al. 2010).

##### Distribution

It is distributed in North Africa, central and south Europe, Middle East, Turkey, Iran, central-west Asia, central and south Urals (Tolman and Lewington 2008). It can be found in mainland Italy, Sardinia and Sicily (Villa et al. 2009).

##### Notes

Eurycora and migratory species, it lives in open environments such as prairies, alpine pastures and cultivated areas, from the plain to 2000 m a.s.l. Trivoltine species with generations in April-May, June-July and August-September (Paolucci 2013, Villa et al. 2009).

#### Pieris rapae

Linnaeus, 1758

00EF13F8-A2D4-5A8D-B029-AB0CC9324D15

##### Ecological interactions

###### Conservation status

Least Concern for Italian (Balletto et al. 2015), Mediterranean (Numa et al. 2016) and European assessment (Van Swaay et al. 2010).

##### Distribution

European species (Sterry and Mackay 2005). It can be found in mainland Italy, Sardinia and Sicily (Villa et al. 2009).

##### Notes

Eurycora and migratory species very widespread in every environment from the plain to 2300m a.s.l., especially in prairies, cultivated areas and arid meadows. It has four generations in March-April, June, August and September-October (Paolucci 2013, Villa et al. 2009).

#### Satyridae

F6A84B27-294B-5868-95E0-1E90069CCF0D

#### Coenonympha pamphilus

Linnaeus, 1758

FCE31DE9-58A4-5F38-8120-B865FE1AEC00

##### Ecological interactions

###### Conservation status

Least Concern for Italian (Balletto et al. 2015), Mediterranean (Numa et al. 2016) and European assessments (Van Swaay et al. 2010).

##### Distribution

It is distributed in North Africa, Europe, Turkey, Middle East and west Mongolia (Tolman and Lewington 2008). It can be found in mainland Italy, Sardinia and Sicily (Villa et al. 2009).

##### Notes

Widespread in a wide range of habitats, often in arid and flower-rich meadows, mountain pastures and rugged fields from the plain to 2100 m a.s.l. It is also found in peatlands and wet meadows. Bivoltine species with generations in April-May, July-August, sometimes a third in October (Paolucci 2013, Tolman and Lewington 2008, Villa et al. 2009).

#### Maniola jurtina

Linnaeus, 1758

5D7435BD-E9BE-5170-944D-152A3C106549

##### Ecological interactions

###### Conservation status

Least Concern for Italian (Balletto et al. 2015), Mediterranean (Numa et al. 2016) and European assessments (Van Swaay et al. 2010).

##### Distribution

It is distributed in Canary Islands, northwest Africa, Europe, Turkey, north Iran, Kazakhstan, central and south Urals to west Siberia (Tolman and Lewington 2008). It can be found in mainland Italy, Sardinia and Sicily (Villa et al. 2009).

##### Notes

Widely distributed, often abundant; it is common to all types of pasture, such as flowery meadows, grassy slopes, neglected cultivated areas; it is also found along open hedges and wooded edges from the plain to 1500 m a.s.l. Univoltine species flies in June-July (Paolucci 2013, Tolman and Lewington 2008, Villa et al. 2009).

### Orthoptera

#### Acrididae

ABED471D-7065-54C4-BA5B-A63BE5466CB9

#### Acrida ungarica mediterranea

Herbst, 1786

8780FDAF-AB44-5E7D-86CE-E535DE3971A2

##### Ecological interactions

###### Conservation status

Least Concern for European (Hochkirch et al. 2016) and Global assessments (Hochkirch et al. 2016a).

##### Distribution

Mediterranean area and Africa (Fontana et al. 2002). In Italy, it is widespread in the whole of the country (Sicily and Sardinia included), with some discontinuity in northern Italy (Baroni et al. 2018, Iorio et al. 2019, Massa et al. 2012).

##### Notes

It is a thermophilous species, living in all kinds of dry habitats, wet grassland, dunes and wasteland. The adults can be found in summer and autumn (Fontana et al. 2002, Massa et al. 2012).

#### Aiolopus strepens strepens

Latreille, 1804

F5D34B9F-4566-5A14-B168-A1F08BD9FF9F

##### Ecological interactions

###### Conservation status

Least Concern for European assessment (Hochkirch et al. 2016).

##### Distribution

Europe and northern Africa to the Caucasus and Middle East (Fontana et al. 2002). Widespread all over in Italy (Sicily and Sardinia included), also on many small islands (Massa et al. 2012, Iorio et al. 2019).

##### Notes

It is a thermophilous species (Fontana et al. 2002), living in habitats with sparse vegetation like dry grassland, roadside verges and forest clearings. Adults can be found throughout the year, even during warm days in winter, while the highest densities occur in autumn (Massa et al. 2012).

#### Aiolopus thalassinus thalassinus

Fabricius, 1781

2DCE5D5E-4804-558E-AEED-143E421EF520

##### Ecological interactions

###### Conservation status

Least Concern for European assessment (Hochkirch et al. 2016).

##### Distribution

Europe, Africa and Asia (Fontana et al. 2002). In Italy, it is widespread in the whole country (Sicily and Sardinia included), but often discontinuously (Baroni et al. 2018, Iorio et al. 2019, Massa et al. 2012).

##### Notes

It is a hygrophilous species, often living in brackish habitats (Fontana et al. 2002). In Italy, it seems to be in decline (Iorio et al. 2019, Massa et al. 2012).

#### Calliptamus italicus italicus

Linnaeus, 1758

AA02E562-BE58-58B5-809A-2F91997D0A97

##### Ecological interactions

###### Conservation status

Least Concern for European assessment (Hochkirch et al. 2016).

##### Distribution

Widely distributed from Europe to Afghanistan (Massa et al. 2012). In Italy, it is widespread over the whole country (Sicily and Sardinia included), but especially in the northern part (Iorio et al. 2019, Massa et al. 2012). It should be typical of the Po Plain (Massa et al. 2012).

##### Notes

It lives in all kinds of dry open, often rocky habitats, with sparse vegetation. They can build up high population densities and it is one of the most harmful species to agriculture in Italy (Massa et al. 2012).

#### Chorthippus (Glyptobothrus) brunneus brunneus

Thunberg, 1815

9543A91B-F9DF-59D9-9D5C-6D1B95C25DE6

##### Ecological interactions

###### Conservation status

Least Concern for European (Hochkirch et al. 2016) and Global assessments (Bushell and Hochkirch 2014).

##### Distribution

From the Balkan Peninsula and north-eastern Italy (Massa et al. 2012). In Italy, it is widespread all over the country. It is only absent in Sicily (Massa et al. 2012, Iorio et al. 2019).

##### Notes

It lives in open habitats with sparse vegetation, like wasteland, mountain slope, forest clearings and roadside verges. The adults can be found from May to October (Massa et al. 2012).

#### Chorthippus (Chorthippus) dorsatus dorsatus

Zetterstedt, 1821

88BC7951-7635-5F74-ACED-D06E40736238

##### Ecological interactions

###### Conservation status

Least Concern for European assessment (Hochkirch et al. 2016).

##### Distribution

From Europe to Siberia (Massa et al. 2012). In Italy, it is widespread in the north and central mainland; the southernmost records are from the south of Abruzzo. There is one record from Sardinia (Iorio et al. 2019, Massa et al. 2012).

##### Notes

It lives from sea level to the mountains, in dry to moist grassy habitats. It can build large populations. The adults can be found in summer and autumn (Massa et al. 2012).

#### Chrysochraon dispar dispar

Germar, 1834

28B36511-99AC-5B64-93C3-14C5294D03B4

##### Ecological interactions

###### Conservation status

Least Concern for European assessment (Hochkirch et al. 2016).

##### Distribution

Central Europe, Middle East to Siberia (Massa et al. 2012). The actual status of the species in Italy has to be re-evaluated. It is known to be present in the north-eastern area of the country, in mountain areas of Veneto and Friuli-Venezia Giulia (Iorio et al. 2019, Massa et al. 2012).

##### Notes

It lives in moist grassland with high vegetation, in marshy and swampy areas (Massa et al. 2012).

#### Euchorthippus declivus

Brisout de Barneville, 1848

621C946A-8CB0-5D37-AD35-84364DC960EB

##### Ecological interactions

###### Conservation status

Least Concern for European (Hochkirch et al. 2016) and Global assessments (Hochkirch et al. 2016b).

##### Distribution

From Europe to Ukraine (Fontana et al. 2002). In Italy, it is widespread throughout the mainland, with a few records in Sardinia (Iorio et al. 2019, Massa et al. 2012).

##### Notes

It is a xerothermophilus species. It lives in dry, stony and sunny meadows, in clearings and at the edge of the woods. Sometimes, it behaves like a hygrophilous species and inhabits fresh and wet meadows and swampy areas. The adults can be found from July to September (Fontana et al. 2002).

#### Locusta migratoria cinerascens

Linnaeus, 1758

EE1CBDED-4158-5C74-AFF5-D3569139BB6F

##### Ecological interactions

###### Conservation status

Least Concern for European assessment (Hochkirch et al. 2016).

##### Distribution

Southern Europe, Mediterranean area, Africa and Asia (Fontana et al. 2002). In Italy, it is widespread in the whole country (Sicily and Sardinia included), but recently, it is much scarcer in the north (Iorio et al. 2019, Massa et al. 2012).

##### Notes

Lives in wet habitats with high grasses and herbs and sandy soil. Adults can be found in summer and autumn (Fontana et al. 2002, Massa et al. 2012).

#### Mecostethus parapleurus parapleurus

Hagenbach, 1822

6ECBD10B-4372-5250-A447-60B0E5508BC1

##### Ecological interactions

###### Conservation status

Least Concern for European assessment (Hochkirch et al. 2016).

##### Distribution

Central and eastern Europe, widespread from Asia to Siberia. In Italy, it is widespread in the northern part, but rare in the Alpine arc (Iorio et al. 2019, Massa et al. 2012).

##### Notes

The species is typical of wet grassland, where it lives in the high vegetation. The adults can be found from June to October. In Italy, it seems to be in decline (Massa et al. 2012).

#### Oedipoda caerulescens caerulescens

Linnaeus, 1758

EADAB1FE-A741-5045-895F-FE4BB5FCFA0A

##### Ecological interactions

###### Conservation status

Least Concern for European assessment (Hochkirch et al. 2016).

##### Distribution

Europe, North Africa, Middle East and Asia to China (Massa et al. 2012). In Italy, it is widespread in the whole country. It is absent from Sardinia (Iorio et al. 2019, Massa et al. 2012).

##### Notes

It lives on bare ground in a wide variety of dry, open habitats like grassland, slopes, roadside verges and forest clearings. The adults can be found in summer and autumn (Massa et al. 2012).

#### Omocestus (Omocestus) rufipes

Zetterstedt, 1821

C07A572D-52B2-50BA-B41E-9BE62E8AD6E2

##### Ecological interactions

###### Conservation status

Least Concern for European assessment (Hochkirch et al. 2016).

##### Distribution

Wide distribution, from Europe and North Africa (Fontana et al. 2002). In Italy, it is widespread over the whole country (Sicily and Sardinia included) (Iorio et al. 2019, Massa et al. 2012).

##### Notes

It lives from the sea level to 2300 m in the mountains, in grasslands, wasteland, forest clearings, urban and agricultural habitats. This species has two generations in one year and can be found from May to November. It is one of the few species living in the Padanian Plain, in cultivated fields, too (Fontana et al. 2002, Massa et al. 2012).

#### Pezotettix giornae

Rossi, 1794

88EE94AA-B339-5522-AFF0-945EFC87C7C4

##### Ecological interactions

###### Conservation status

Least Concern for European assessment (Hochkirch et al. 2016).

##### Distribution

It is distributed in south Europe, North Africa as far east as Caucasus (Fontana et al. 2002). In Italy, it is widespread in the whole country (Sicily and Sardinia included), also on several small islands (Iorio et al. 2019, Massa et al. 2012).

##### Notes

It is a thermophilus species and lives in many different open habitats. It lives in meadow environments, preferably mesoxerophilous; it can frequent mountain stony and arid stony environments. The adults appear from June to October, but it is frequent to observe them in autumn during the mating season (Fontana et al. 2002, Massa et al. 2012).

#### Pseudochorthippus parallelus parallelus

Zetterstedt, 1821

EBA2E88D-F5B5-5C00-863D-229A97024006

##### Ecological interactions

###### Conservation status

Least Concern for European assessment (Hochkirch et al. 2016).

##### Distribution

It is distributed from Europe to Siberia. In Italy, it is widespread in the Alps and Apennines. From Sardinia, there is a doubtful record (Fontana et al. 2002).

##### Notes

It is a mesohygrophilus to hygrophilus montane species. The adults can be found in summer and autumn in dry to moist grassland (Fontana et al. 2002).

#### Tetrigidae

EDFBB433-EB6E-571D-94A5-7A761853BFC1

#### Tetrix subulata

Linnaeus, 1758

4B634233-6B7C-5D50-83A4-CD307476BCA6

##### Ecological interactions

###### Conservation status

Least Concern for European assessment (Hochkirch et al. 2016).

##### Distribution

Widely distributed species in Eurasia and North America (Fontana et al. 2002). In Italy, it is recorded in many localities in the mainland, while with single records in Sicily and Sardinia (Iorio et al. 2019, Massa et al. 2012).

##### Notes

It is a meso-hygrophilous or hygrophilous species and lives from the coast up to 1700 m a.s.l. More frequent in lowland areas, submontane up to 1000 m a.s.l. It often forms abundant populations, located in fresh and humid habitats. It is an early species that overwinters as a nymph or adult insect (Fontana et al. 2002).

#### Tetrix tenuicornis

Sahlberg, 1893

45408A57-2A41-5E3F-AF9C-43582995AAAC

##### Ecological interactions

###### Conservation status

Least Concern for European assessment (Hochkirch et al. 2016).

##### Distribution

Central and east Europe and Middle East. In Italy, it is widespread in the north-eastern regions, scarce in the north-west and with some records in the central Apennines (Iorio et al. 2019, Massa et al. 2012).

##### Notes

It is a small meso-hygrophilous species. It is common from the coast to submontane habitats, up to 1100 m a.s.l (Fontana et al. 2002). It forms abundant population in the slightly moist parts of dry grassland, along irrigation canals or in irrigated meadows. The adult can be found throughout the year, even if the species probably winters as a nymph (Massa et al. 2012).

#### Tettigonidae

8631DEB1-9912-5474-A735-945CFBFD3131

#### Platycleis grisea grisea

Fabricius, 1781

F758511F-371D-5AFA-806B-CE1069267120

##### Distribution

Central, southern and eastern Europe to southern Russia. In Italy, it is widespread over the whole country, most abundant in the northern regions. In Sicily, it is known only from Etna (Iorio et al. 2019, Massa et al. 2012).

##### Notes

It is a widespread species. It lives from sea level to high up in the mountains, in dry open habitats, mostly in patches with dense vegetation. The adults can be found in summer and autumn (Massa et al. 2012).

#### Roeseliana azami azami

Finot, 1892

6360AB4A-CC0A-5C6C-A5EE-8FFE44588611

##### Ecological interactions

###### Conservation status

Vulnerable for European (Hochkirch et al. 2016), Mediterranean and Global assessments (Braud et al. 2016).

##### Distribution

Endemic species of southern France, recently reported in Italy (Bardiani and Buzzetti 2009). A review of the genus is underway (Iorio et al. 2019).

##### Notes

In France, it lives in wet meadows and along the edges of streams. In Italy, it is reported as a mountain and hygrophilous species. It lives in wet grasslands, in dense vegetation. The adults can be found in summer and autumn (Massa et al. 2012).

#### Ruspolia nitidula

Scopoli, 1786

515CBC5C-F436-52F8-9C3F-3000CF039755

##### Ecological interactions

###### Conservation status

Least Concern for European assessment (Hochkirch et al. 2016).

##### Distribution

Widespread species, from central-southern Europe to Palearctic Asia and Africa (Fontana et al. 2002). In Italy, it is widespread all over the country (Sicily and Sardinia included) (Iorio et al. 2019, Massa et al. 2012).

##### Notes

Meso-hygrophilous or hygrophilous species, it inhabits all kinds of open habitats with a high rate of humidity and dense vegetation. It is frequent in agricultural fields, grasslands, banks of rivers, lakes and canals and also in brackish wetlands. It also lives in urban environments (Fontana et al. 2002, Massa et al. 2012).

#### Tettigonia viridissima

Linnaeus, 1758

ACB0B5D5-E260-5168-A31B-128D3EF895FD

##### Ecological interactions

###### Conservation status

Least Concern for European assessment (Hochkirch et al. 2016).

##### Distribution

Holopaleartic species, widespread in Europe and north-western Africa. In Italy, it is widespread in the whole country (Siciliy and Sardinia included) (Iorio et al. 2019, Massa et al. 2012).

##### Notes

Mesophilic species with a wide ecological value. It lives from sea level up to higher elevations. It can be found among the tall grasses of the meadows, on bushes and on the crown of trees. It feeds on various insects and larvae and vegetarian nutrition is limited. Adults can be found in summer and autumn (Massa et al. 2012).

## Analysis

### Data analysis

Statistical analysis was conducted exclusively on ground beetles, rove beetles and spiders sampled by pitfall traps because only these taxa had a sufficient amount of quantitative data available. For spiders and beetles, immature specimens and larvae, respectively, were not considered in the analysis because, for many of them, it was not possible to determine the species. For the other groups, only descriptive tables have been prepared.

To examine whether trap locations were sufficiently spaced to be independent replicates, we tested our data for autocorrelation by performing a Mantel test, based on Pearson’s product-moment correlation (permutations: 9999) between Bray-Curtis distances in assemblage composition and the geographical distances of samples collected. We found that spatial correlation in assemblages between samples was low (Person’s r = 0.43) and not significant (p ≥ 0.05). Therefore, we assumed all sampling plots as statistically independent (inter-sample distance ≥ 10 m). From hereafter, each trap is called a plot and each field (with six plots) a site.

The mean number of species and individuals per plot was calculated and the difference in species richness amongst sites was evaluated with the Kruskal–Wallis non-parametric analysis of variance. Differences amongst individual factor levels was tested by Wilcoxon pairwise rank sum tests.

Differences in species richness amongst sites was also evaluated by computing a sample size-based rarefaction curve using the software iNEXT (interpolation/extrapolation) R package (Hsieh et al. 2016). iNEXT focuses on three measures of Hill numbers (Hill 1973) of order q: species richness (q = 0: the relative abundances of species are not considered and, therefore, the value obtained is equal to that of species richness), Shannon diversity (q = 1: the index weighs the species, based on their frequency and the result is the exponential version of the Shannon Index) and Simpson diversity (q = 2: the abundant species have a higher weight and the result is the inverse of the Simpson concentration).

For each diversity measure, iNEXT uses the observed sample of abundance data to compute diversity estimates, calculated via the functions ChaoRichness for q = 0, ChaoShannon for q = 1 and ChaoSimpson for q = 2; (see Chao et al. 2014 for the formulae of these asymptotic estimators). The 95% confidence intervals associated with the estimates are also calculated and a sample-size-based rarefaction and extrapolation (R/E) curve is plotted.

In order to verify differences in species composition amongst the *marcita* fields, we performed a PERMANOVA (permutational multivariate analysis of variance) analysis, using Primer 6+ statistical software with the PERMANOVA + add-on package (Anderson et al. 2006, Clarke and Gorley 2006). The analysis was conducted on a Bray–Curtis similarity matrix in which abundance raw data were standardised by dividing the number of individuals of each species collected in each plot by the total number of individuals collected in that plot. These data were then square-root transformed to underestimate the contribution of the more abundant species (Clarke 1993, Bray and Curtis 1957). Pairwise post-hoc tests to compare similarities in species composition amongst sites were performed under 9999 permutations (for further details, see Anderson 2005). Whenever the sample size was too small once divided into factors, PERMANOVA's Monte Carlo test, which uses chi-square variables, combined with eigenvalues to construct the asymptotic permutation, was used.

We also used the Bray–Curtis similarity matrix in a distance-based Redundancy Analysis – dbRDA (Anderson and Cribble 1998, Ramette and Tiedje 2007) to display differences in species composition amongst all samples and the contribution of individual species to these differences. The contribution of each species in determining the dissimilarity between pairs of *marcita* fields was measured using the SIMPER test (Anderson et al. 2006, Clarke and Gorley 2006) which provides a percentage measurement of this contribution according to a decreasing dissimilarity order.

### Results

During the whole sampling period we found a total of 47 ground beetle species, 35 rove beetle species, 29 spider species, one Lucanidae, 16 butterfly species and 24 grasshopper and cricket species. Specifically, between April and October 2014, we found a total of 4449 ground beetles belonging to 41 species (Table 2); 1698 spiders belonging to 29 species (Table 3); 589 rove beetles belonging to 34 species (Table 4); 45 butterflies belonging to 16 species (Table 5). During the winter 2014/2015, we collected 618 overwintering beetles belonging to 22 species divided as follows: 17 ground beetle species, four rove beetle species and one species belonging to Lucanidae (Table 6).

Between May and September 2015, we found a total of 262 grasshoppers and crickets belonging to 24 species (Table 7) and we confirmed the presence of *Lycaena dispar* at the "Amerio 2" field in the Sforzesca area.

**Ground beetle richness and abundance**

During the summer of 2014, carabid assemblage in the whole study area was dominated by macropterous individuals (4331 out of 117, for a total of 38 species), both omnivorous and predators (Table 2). We found only two brachypterous species, Calathus (Neocalathus) melanocephalus and *Microlestes minutulus*, both predators, for a total of five individuals and one dimorphic species, Carabus (Carabus) granulatus (n = 112), which is also a predator. Amongst the collected ground beetles, we found only one phytophagus species: *Diachromus germanus.* The most abundant species was Pseudoophonus (Pseudoophonus) rufipes with more than 1000 specimens, followed by Metallina (Metallina) properans and Harpalus (Harpalus) affinis with more than 500 specimens (Table 2).

**Figure 2.**
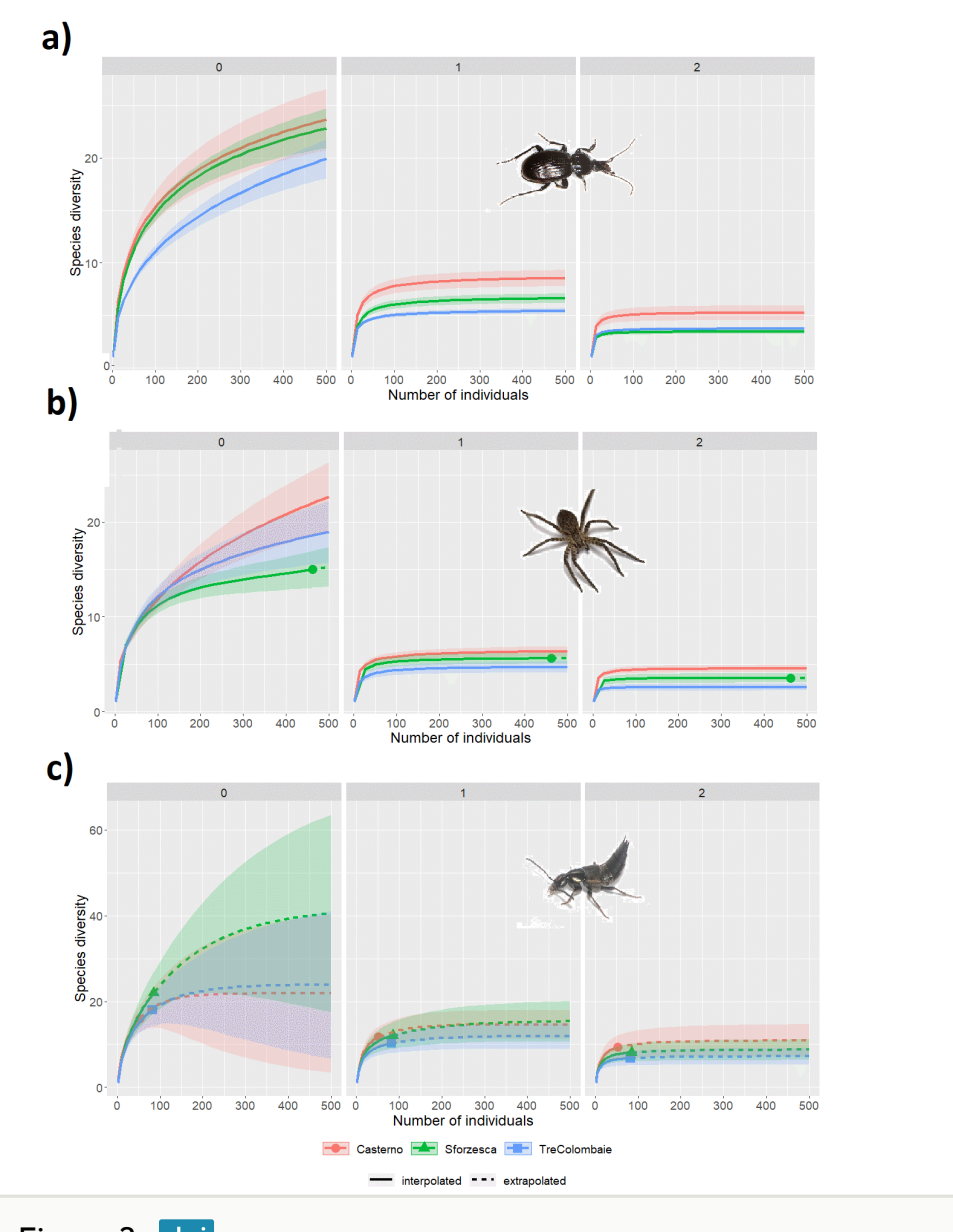
Rarefaction curves showing the diversity of a) ground beetles; b) spiders; c) rove beetles, in the three *marcita* fields when the order q was set at q = 0 (species richness), q=1 (exponential Shannon index) and q=2 (Simpson index).

Ground beetle species richness differed significantly amongst the three *marcita* fields (Kruskal-Wallis chi-squared = 8.68, df = 2, p-value = 0.013). The field with the highest number of species was Tre Colombaie, followed by Sforzesca and Casterno (Table 8). Although Casterno resulted in the field with the lowest number of species (Wilcoxon rank sum test: Casterno vs. Tre Colombaie, p = 0.024; Casterno vs. Sforzesca p = 0.036), it showed a more equitable species abundance compared to the other two fields. Therefore, as shown in Fig. 2, Casterno is the field with the highest Shannon and Simpson diversity.

The PERMANOVA test revealed differences in species composition amongst the fields (Table 9). In particular, Casterno was significantly different from Sforzesca and Tre Colombaie (Table 10) showing a low frequency of some species, such as Pseudoophonus (Pseudoophonus) rufipes and Harpalus (Harpalus) affinis, that were otherwise significantly abundant in the other two fields (Table 2). Species composition differed also between Sforzesca and Tre Colombaie (Table 10). The species that contributed the most to the dissimilarity between the two fields was Harpalus (Harpalus) affinis, which was clearly more abundant in Tre Colombaie (Table 11).

**Figure 3.**
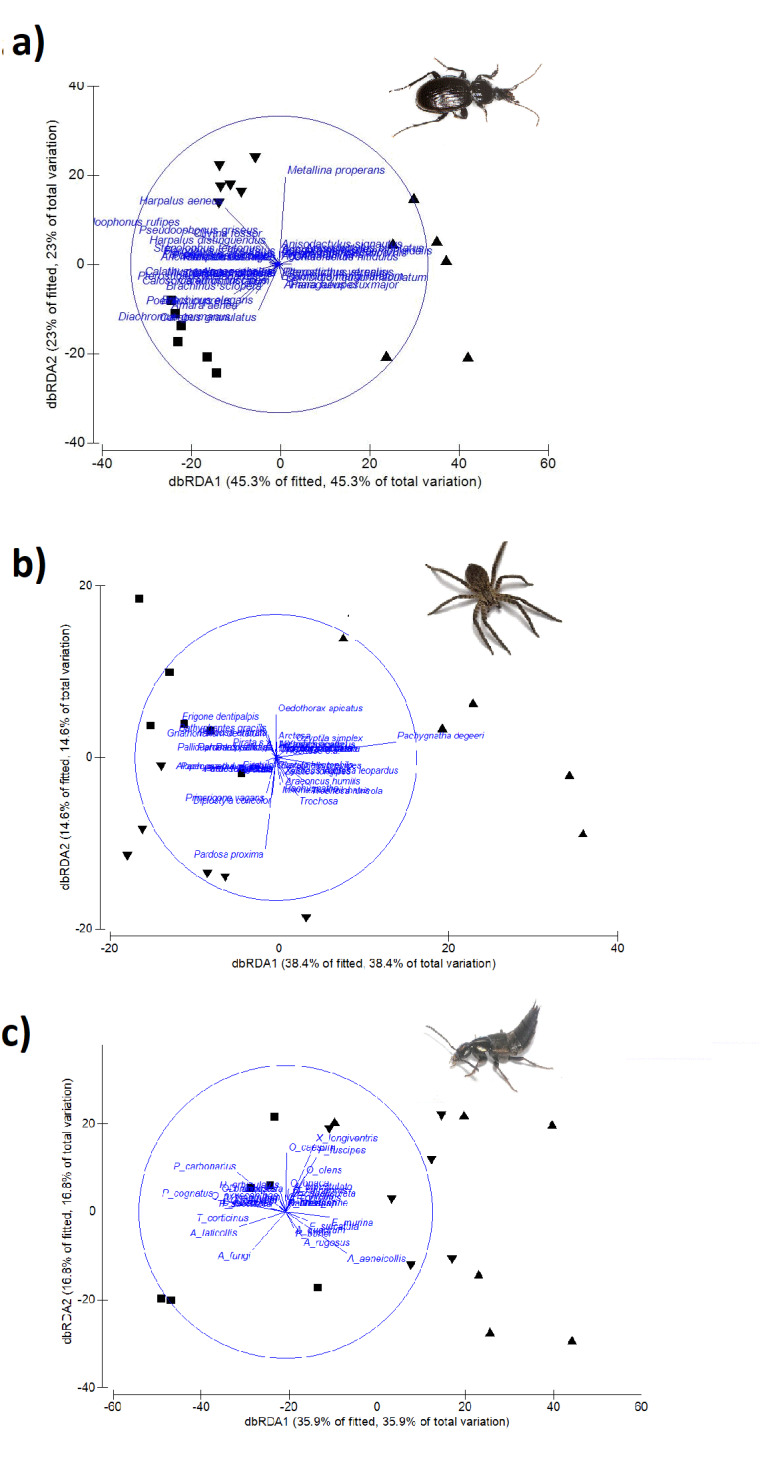
Distance-based redundancy analysis (dbRDA) ordination plot of ground beetle, spiders and rove beetle assemblages in the three *marcita* fields (Casterno: triangle; Tre Colombaie: inverted triangle; Sforzesca: square)

Ordinations with db-RDA confirm differences in species composition amongst the three communities (Fig. 3). In particular, the first axis explains 45% of the total variance and clearly separates Casterno from Sforzesca and Tre Colombaie ground beetle communities. The second axis, with 23% of the total variance explained, separates Sforzesca from Tre Colombaie communities. In the cloud of species projected in the db-RDA graph, also less frequent species are present. Amongst them, those collected only on one occasion are: Harpalus (Harpalus) serripes and Pterostichus (Melanius) aterrimus in the Tre Colombaie field, Bembidion (Bembidion) quadrimaculatum, *Sphaerotachys hoemorrhoidalis* and Stenolophus (Egodroma) marginatus in the Casterno field, Parophonus (Parophonus) maculicornis and Amara (Amara) similata in the Sforzesca field.

**Spider richness and abundance**

The whole spider community analysed has 1698 specimens with a slightly unbalanced sex ratio in favour of males (1019 vs. 763 females). *Pardosa proxima* (Lycodidae) was the most abundant species with 731 specimens mainly sampled in Tre Colombaie (Table 3), followed by *Pachygnatha degeeri* (Tetragnathidae), with 251 specimens mainly sampled in Casterno, *Oedothorax apicatus* (Linyphiidae) with 226 specimens equally distributed amongst the fields and *Arctosa leopardus* (Lycosidae) with 188 specimens mainly sampled in Casterno (Table 3).

Spider richness ranged from 25 species in Casterno to 19 and 15 species in Tre Colombaie and Sforzesca, respectively (Table 12). The higher diversity value of the Casterno community remains constant as the order q changes (Fig. 2). Conversely, Tre Colombaie and Sforzesca showed, respectively, a decrease and an increase in the diversity value as the order q increased. The mean species richness did not differ significantly amongst the three *marcita* fields (Kruskal-Wallis chi-squared = 5.48, df = 2, p-value = 0.065). However, the higher number of species, found in Casterno, makes this value very close to significance.

Species composition differed significantly amongst the fields (Table 9). Similarly to ground beetles, spider species sampled in Casterno were also very different from those sampled in Tre Colombaie and Sforzesca (Table 10). Casterno is characterised by the presence of a very abundant species, the Tetragnathidae *Pachygnatha degeeri*, which is almost completely absent in the other two fields. This species contributes to 21.4% of the dissimilarity between Casterno and Tre Colombaie and 21.7% of the dissimilarity between Casterno and Sforzesca (Table 11). Conversely, species composition of Tre Colombaie and Sforzesca were similar.

Ordinations with db-RDA confirm differences in species composition amongst the three communities also highlighting the species that contribute most to this difference (Fig. 3). The first axis explains 38.4% of the variance (53% total variance from the first two axis) and clearly separates Casterno from Sforzesca and Tre Colombaie spider communities. The second axis, with 14.6% of total variance explained, separates Sforzesca from Tre Colombaie communities. In the cloud of species projected in the db-RDA graph,10 species sampled exclusively in Casterno and three species sampled exclusively in Tre Colombaie are also shown (Table 3).

**Rove beetle richness and abundance**

During summer 2014, rove beetle richness ranged from 21 species in Tre Colombaie to 18 and 16 species in Sforzesca and Casterno, respectively (Table 13). Although there was a high number of species in Tre Colombaie, the mean species richness did not differ significantly amongst the three *marcita* fields (Kruskal-Wallis chi-squared = 1.21, df = 2, p-value = 0.545). Moreover, rove beetle communities from Casterno and Sforzesca fields showed very similar values of Shannon and Simpson indices (Table 13, Fig. 2).

Philonthus (Philonthus) carbonarius was the most abundant species with 172 specimens sampled mainly in Tre Colombaie and Sforzesca. The second most abundant species, Atheta (Atheta) aeneicollis, with 129 specimens, was sampled mainly in Tre Colombaie and Casterno (Table 4).

The PERMANOVA test revealed differences in species composition amongst the three fields (Tables 9, 10). Ordinations with db-RDA confirmed differences in species composition amongst the three communities (Fig. 3) and the first axis, explaining 39.1% of the total variance, clearly separated Sforzesca from Casterno and Tre Colombaie rove beetle communities. In the cloud projected in the db-RDA graph, the species that characterises each community is clearly visible. The species that contributed the most to dissimilarity between fields was Philonthus (Philonthus) cognatus, contributing to 15.5% of the dissimilarity between Sforzesca and Tre Colombaie and 15.8% of the dissimilarity between Sforzesca and Casterno (Table 11).

**Butterfly richness and abundance**

Amongst butterfly families, the most representative one was that of Pieridae with three species (Table 5). The most abundant species belonging to this family was *Pieris rapae*, which is found mainly in Sforzesca. All the other families had two species with the exception of Hesperiidae with only one member. Tre Colombaie was the most diversified field with at least one species per family and four exclusive species: *Pyrausta despicata*, *Pyrgus* sp., *Autographa gamma* and *Vanessa cardui*, while Sforzesca was the leastdiversified one with only five families out of eight. However, this was the only field where two Lycaenidae are present: *Lycaena dispar* (also sampled in the other two fields) and *Lycaena phlaeas* (Table 5). Finally, the species *Colias alfacariensis* and *Loxostege sticticalis* were found exclusively in Casterno.

**Overwintering beetle richness and abundance**

The largest number of collected species during winter 2014-2015 belongs to the ground beetle family (Carabidae) (Table 6). Carabidae was also the most abundant family found in Casterno and Tre Colombaie. Within this family, Carabus (Carabus) granulatus, Panagaeus (Panagaeus) cruxmajor and Anchomenus (Anchomenus) dorsalis were the most abundant species with 63, 39 and 24 specimens, respectively (Table 6).

Staphilinidae was the most abundant family found in Sforzesca. Amongst rove beetles (four species), the most abundant was Paederus (Heteropaederus) fuscipes with 408 specimens, almost all collected in Casterno (Table 6).

The other collected family has only one representative: *Dorcus parallelepipedus* (Lucanidae) with only two specimens found exclusively in Tre Colombaie.

**Grasshopper and Cricket richness and abundance**

Amongst the grasshoppers and crickets, the most representative family was Acrididae with a number of species much higher than the other families (Table 7). The most abundant species were the Acrididae *Pseudochorthippus parallelus* with 55 specimens and *Aiolopus thalassinus* with 48 specimens (Table 7).

Concerning ecological traits, the orthopteran assemblage was dominated by highly mobile species (11 out of 20 identified species), followed by eight intermediate dispersers species and one sedentary species, *Roeseliana azami*. Medium specialised species represent a large proportion of the community observed in the winter-irrigated meadows investigated (n = 10), while habitat specialist (n = 5) and generalist (n = 5) orthopterans were less common.

## Discussion

**Ground beetle community**

Most of the beetle individuals sampled in *marcita* fields during the summer of 2014 were macropterous (i.e. with high dispersal ability) and omnivorous, adapted to living in periodically-disturbed sites, such as watercourses or grasslands (Rainio and Niemelä 2003). In perturbed habitats, species face an elevated risk of local extinction and the ability to relocate by flight to new favourable patches when resource availability suddenly changes is essential to survival (Ribera et al. 2001, Gobbi and Fontaneto 2008). Moreover, omnivorous species, due to their wide trophic niche breadth and great resilience to reductions in food supply, better persist in stochastic environments (Purtauf et al. 2004, Schweiger et al. 2005).

However, we also found two brachypterous, Calathus (Neocalathus) melanocephalus and *Microlestes minutulus*, one dimorphic, Carabus (Carabus) granulatus and a conspicuous number of predatory species (23 out of 41, constituting 56% of the collected individuals). The presence of these species confirms that traditional agricultural habitats, such as *marcita*, could contribute to the persistence of individuals with low dispersal ability and also of strictly predatory species in intensive agroecosystems (Brandmayr et al. 2005, Cardarelli and Bogliani 2014).

Even if we did not find any endemism, we recorded the presence of *Dolichus halensis*, that has been identified as a focal species of wet meadows of the Po Plain (Bogliani et al. 2007). We also unfortunately confirmed the disappearance from the investigated *marcita* fields of Carabus (Limnocarabus) clatratus antonellii, documented as closely associated with this biotope (Bucciarelli 1963) and extinct in the Ticino Park for over 30 years, despite the apparent persistence of the habitat that was considered suitable.

Casterno was the field with the lowest number of species, but with the highest Shannon and Simpson diversity. All the species found in Casterno were equitably distributed showing this site to have more stable environmental conditions (Death 1996) compared to the other two investigated fields. Contrarily, Sforzesca and Tre Colombaie communities are characterised by few and abundant species resulting in communities that are very different from those of Casterno. In particular, the PERMANOVA highlighted the presence of two very abundant species, Pseudoophonus (Pseudoophonus) rufipes and Harpalus (Harpalus) affinis, which, respectively, characterise Sforzesca and Tre Colombaie fields. Both these species are clearly dominant within the family Carabidae occurring in agro-ecosystems and are widely considered generalist species with a broad ecological valence (Porhajašová et al. 2014).

**Spider community**

Collected spiders were all very common and quite frequent (Nentwig et al. 2020), with the only exception being *Ceratinella brevipes*, a rare, detrital species that lives both in woodlands and grasslands (Hansen 2010). Half of the species was linked to wet environments, but only two of them can be considered stenohygrophilous: *Gnathonarium dentatum* (Linyphiidae) and *Pirata tenuitarsis* (Lycosidae) (Hansen 2010).

Both Linyphiidae and Lycosidae were very abundant in this study. Linyphiidae are the most abundant spiders in temperate regions (Samiayyan 2014) and this is probably the main reason why they were so frequently captured in our samples. Moreover, the use of pitfall traps can partially explain the abundance of Lycosidae, as this family includes species living and hunting on the ground and so easily interceptable by pitfall traps (Green 1999). Both these families are also typical of highly dynamic ecosystems, especially those disturbed by frequent flooding (Buchholz and Schröder 2013), as *marcita*. Many Lycosidae, such as *Arctosa leopardus* and *Pardosa proxima* s.l., recorded in high numbers in our fields, are specialised for living in wet habitats with temporal flooding, while Linyphiidae are able to rapidly recolonise disturbed areas and survive flooding through ballooning (Hayashi et al. 2015, Holland et al. 2007).

Most of the collected individuals were males. Indeed, males, because of their mate-searching behaviour (Lang 2000, Collins et al. 1996), are more mobile and, therefore, more easily intercepted by traps.

Concerning the spider community in the three *marcita*, Casterno resulted in being significantly different from the other two fields. In Casterno, spiders species, belonging to Lycosidae and Linyphiidae, were almost double those found in Tre Colombaie and Sforzesca, making this field the richest in species. Moreover, rarefaction curves showed higher species diversity and a more equitable distribution of the species abundance in Casterno compared to the other two fields where few, very abundant species were found. PERMANOVA and db-RDA also showed how Casterno hosted a well-characterised community that greatly differs from those of the other two fields. Casterno was the only field to host the rare *Ceratinella brevipes* and the stenohygrophilous *Pirata tenuitarsis*. Moreover, in this field, some hygrophilous species, such as *Pachygnatha degeeri*, *Arctosa leopardus* and *Trochosa ruricola*, were very abundant.

On the other hand, analyses showed a very poor spider community in Sforzesca and Tre Colombaie. Most of the species collected in the two fields, including the exclusive captures, such as *Palliduphantes pallidus* and *Ozyptila sanctuaria*, were less related to wet environments and, therefore, in general, less indicative of this type of habitat.

**Rove beetle community**

All the rove beetles sampled during the summer of 2014 were good flyers of open habitat, with wide distribution and ecological value. The only exception was Ocypus (Matidus) brunnipes, a flightless species that inhabits both forested and open disturbed areas (Deichsel 2006). Only *Ochthephilum brevipenne* was exclusively linked to wet habitat, as it is usually found in ponds, along watercourses, in marshy meadows and near the seashore (Bordoni et al. 2006). All other species can be found also in less humid habitats and one species, the uncommon *Astrapaeus ulmi*, is thermophilous and mainly lives in xerothermic plant communities (Wojas 2011).

Most of the species were predators, linked to meadows or non-cropped areas (Zanetti et al. 2016). Amongst them, Ocypus (Ocypus) olens, recorded in all studied fields even if in low number, is known to be a species sensitive to agricultural practices (Daccordi and Zanetti 1989), highlighting the lower pressure to which this traditional agricultural habitat undergoes with respect to conventional, intensive crops. According to what was found for ground beetles, *marcita* may act as a refuge from detrimental agronomic practices for more sensitive species (Bordoni et al. 2006, Wojas 2011).

The number of rove beetle species was very similar amongst the three *marcita*. Most of the species were shared between fields and, according to the rarefaction curves, species abundance of the three communites are equally distributed. db-RBDA showed a less clear distinction amongst the rove beetles of the three fields compared to what was observed for spiders. However, PERMANOVA analyisis revealed the existence of a different species composition amongst the three *marcita* fields. About 20% of the species was found exclusively in one of the three fields. In particular, the flightless Ocypus (Matidus) brunnipes was collected only in Casterno, while the hygrophilous *Ochthephilum brevipenne* and the thermophilous *Astrapaeus ulmi* were exclusively found in Tre Colombaie. Again, Casterno seems to host species more typical of humid and well-preserved habitat compared to those more common and generalist collected in Tre Colombaie and Sforzesca. Amongst the commonest species shared between Sforzesca and Tre Colombaie, there is Philonthus (Philonthus) carbonarius, a species related to anthropogenic habitats (Lupi et al. 2006).

**Butterfly community**

Most of the sampled species are included in the European and Italian IUCN Red List as "Least Concern" (Balletto et al. 2015, Bonelli et al. 2018, Van Swaay et al. 2010). Particularly important is the presence of *Lycaena dispar* in all the investigated sites. This butterfly is the only globally-recognised one as "Near Threatened" in our study area (Gimenez 1996). The species is an indicator of the importance of *marcita* as a humid habitat in the Ticino Valley Regional Park and, again, the important role that this traditional agricultural habitat may have in preserving specialised, sensitive species. Indeed, *Lycaena dispar* is of great conservation interest as it is included in Annexes II and IV of the Habitats Directive 92/43/CEE and listed in Appendix II of the Berne Convention. It is typical of lowland humid areas, such as meadows, canals and river banks with slow courses. It is in decline in most of its range due to degradation and destruction of its environment, which is threatened mainly by conversion to other uses of open wetlands. Amongst the conservation strategies suggested by the IUCN for the conservation of this species, there are, first and foremost, the protection of reproductive sites avoiding excessive off-season mowing and radical cleaning of ditches and canal banks (Thomas et al. 2011). The importance of *Lycaena dispar* presence in the area and of the maintenance of *marcita* fields is also highlighted by local studies. *Lycaena dispar* has been identified as a focal species of wet meadows of the Po Plain and described as “rare, localized, of conservation interest and indicator of the habitat” (Bogliani et al. 2007).

**Grasshopper and cricket community**

The orthopterans of the *marcita* fields, as well as for the other investigated taxa, reflected the high dynamism of this biotope, characterised during the summer by the succession of flooded and dry phases. Indeed, most of the sampled species had a high dispersal capacity, a typical feature of arthropods adapted to temporary and perturbed habitats ([Bibr B6007897][Bibr B5894481]).

Orthopteran communities composed of a significant percentage of mobile species were also described for rice agro-ecosystems in the western Po Plain, (Giuliano and Bogliani 2018). Moreover, as in rice agro-ecosystems, *marcita* hosted some thermophilous species probably favoured by the presence of xerothermic micro-habitats, such as bare banks, mown levees and unpaved country roads surrounding the fields. During the summer, the alternation of flooded and dry phases could favour the co-existence of thermophilous and hygrophilous elements.

According to the IUCN European Red List of Orthopterans (Hochkirch et al. 2016), most of the species observed in the winter-flooded meadows are considered of least conservation concern. Amongst them, three species, *Euchorthippus declivus*, *Chorthippus Bunneus* and *Acrida ungarica*, were globally assessed as "Least Concern" (Hochkirch et al. 2016). However, it is important to remark on the presence of the hygrophilous *Roeseliana azami*, assessed as "Vulnerable" in Mediterranian regions, in Europe and throughout the world (Braud et al. 2016) and of *Mecostethus parapleurus* and *Chrysochraon dispar*. *Mecostethus parapleurus* is typical of humid environments, such as swamps, water meadows and peat bogs and is threatened and rare in the Italian territory (Tami et al. 2005, Baroni 2015). *Chrysochraon dispar* is a hygrophilous and stenotherm species inhabiting marshes, swamps, wet meadows and brackish biotopes (Nadig 1986). It is a rare and protected species in France, Germany, Austria and Switzerland (Detzel 1998). In Italy, the degree of threat for this species is still unknown, but it is probably at risk of extinction (Tami et al. 2005). Currently, *Chrysochraon dispar* has been found only in Trentino and Alta Val Venosta, with the subspecies *C. dispar dispar* (but here could already be extinct), while in Veneto, in Friuli-Venezia Giulia and in Venice Lagoon, with the subspecies *C. dispar giganteus* (Tami et al. 2005). In the Lombardy Region, the species was recently found in the Ticino Valley Regional Park (Roberto Scherini, pers comm). The maintenance and, where possible, the re-naturalisation of wet areas is considered essential to guarantee the species survival (Tami et al. 2005).

Again, as already pointed out for the other investigated taxa, the presence of extensively-managed habitats in agro-ecosystems, such as *marcita*, may also provide refuge to species more sensitive to human disturbance (Giuliano et al. 2018).

**Overall arthropod community in the investigated fields**

Overall, the winter-flooded meadow system of our study area hosted a rich and diverse entomofauna with indicator species of a hygrophilous environment, such as the butterfly *Lycaena dispar*, the ground beetle *Dolichus halensis* and the grasshopper *Chrysochraon dispar*. However, the arthropod communities of the investigated fields differed from each other in terms of site specificity, species richness and species composition.

In particular, Casterno field stands out for having a high number of exclusive species in all taxa, including two spiders of high conservation value, such as *Ceratinella brevipes* and *Pirata tenuitarsis*. Tre Colombaie field is undoubtedly the richest both for the presence of a large number of species in all taxa and to host exclusive, “priority” species, that are also indicators of a wet environment, such as *Dolichus halensis*. Sforzesca field, although neither stands out for species richness nor for the presence of exclusive species, also hosts specialised species and species of conservation relevance, such as the butterfly *Lycaena dispar*, the two brachypterous ground beetles *Calathus melanocephalus* and *Microlestes minutulus* and the stenohygrophilous spider *Gnathonarium dentatum*. However, these are also species found in the other two fields, so they do not give Sforzesca a uniqueness.

There are no differences in the agronomic management of the three *marcita* fields. In all of them, the winter flooding has been practised for at least five years and mowing has been carried out 3-4 times during the summer season. The only field that has been periodically fertilised is Casterno, but taking into account the high degree of diversity found in the three *marcita*, fertilisation is probably not a determining factor influencing the biodiversity. Likely, the differences amongst sites is found in the landscape diversity, as the mosaic of habitat surrounding the field (e.g. hedgerows, old isolated trees, small woodlots) could determine the facility with which specialised species could colonise the *marcita* fields at first and then after disturbing events, such as the periodic mowing and flooding.

On the other hand, the practice of winter flooding could be considered crucial to guaranteeing the presence of species of wet habitats. In fact, amongst the *marcita* investigated in 2015, the only one to be completely devoid of hygrophilous species was the Amerio 1 which, until 2013, was subjected to total abandonment and in which the winter flooding was carried out exclusively during the winter 2014-2015. The situation was slightly better in Amerio 2, where the meadow management resumed a year earlier than Amerio 1 and lasted until 2016. This certainly favoured the presence of *Lycaena dispar*.

The only field investigated in 2015, in which a winter flooding was comparable to that of Casterno, Sforzesca and Tre Colombaie was carried out, was at “Garlaschè” field. It is the only one in which *Chrysochraon dispar*, a rare and strongly indicative species of wet environments, has been observed.

### Conclusion

This study represents the first comprehensive contribution to the knowledge of terrestrial arthropod communities associated with the winter-irrigated meadows, the so-called *marcita*, in Europe. Our investigation showed that *marcita* fields hosted specialised species (e.g. brachypterous, predators) and species typical of hygrophilous habitats. This result confirms the importance of *marcita* for invertebrate conservation, as already stated for other taxa, such as amphibians and birds (Casale 2015, Casale et al. 2016, Gentilli et al. 1997). It also highlights the importance for biodiversity conservation, to preserve traditional and extensive crops as refuge habitats in highly intensive and exploited agroecosystems, such as that of the fertile Po Plain in Italy (Casale et al. 2017).

Moreover, *marcita* plays a significant role in terms of the ecosystem services provided: they may be a source of bio-controllers through their important bulk of predatory species detected amongst carabids, rove beetles and spiders, that could move to surrounding fields, contributing to limit crop pests (Nyffeler and Sunderland 2003, Symondson et al. 2002). Pest regulation has been demonstrated to be strengthened by complementarity amongst natural enemies, guaranteed by a various guild of natural enemies (Dainese et al. 2017).

Finally, *marcita* has an undeniable aesthetic value, as they are considered a “Rural traditional landscape of Italy” by MIPAAF (Bove et al. 2017), contributing to increasing the landscape beauty of the area.

Since the late eighties, the Ticino Valley Regional Park is making a huge effort in terms of incentives to farmers to preserve and restore these winter-flooded meadows (Casale et al. 2016), together with events to bring back the cultural and historical value of this crop to the attention of local people. A plan to support the re-activation of a sustainable production chain, aiming to re-introduce this high-quality forage in the local zootechnical practices (Bove et al. 2016) and sensitise the consumers on the quality of dairy products obtained from green forage (Elgersma et al. 2006) would certainly help to increase the attraction of this crop amongst farmers.

Obviously, the work to be done is still demanding. A more synergic conservation effort amongst local and regional managing bodies is desirable to restore, where possible, this precious habitat and to retain the biodiversity linked to it.

## Figures and Tables

**Table 1.** Coordinates (latitude and longitude in datum WGS84) of the six investigated *Marcita* fields and their altitude.

***marcita***	**Latitude / Longitude**	**Altitude (m a.s.l.)**
Tre Colombaie	45.309175, 8.818740	110
Cascina Grande	45.422929, 8.865366	110
Sforzesca	45.284550, 8.901501	90
Garlaschè	8.899698, 45.292460	85
Amerio 1	45.280201, 8.900642	80
Amerio 2	45.278269, 8.897209	95

**Table 2.** List of ground beetle species collected in the three *marcita* fields of the study area during the summer of 2014. For each species, information is reported for wing development WD (M = Macropterous, winged species; D = dimorphic, a species that can be either winged or not winged; B = Brachypterous, species without wings or reduced ones) and diet D (P = Predator, OM = Omnivorous, PHY = Phytophagous).

**Species**	**WD**	**D**	**Field**			**Total**
			**Casterno**	**Sforzesca**	**Tre Colombaie**	
Agonum (Olisares) emarginatum (Gyllenhal, 1827)	M	P	1	1	0	2
Agonum (Agonum) muelleri (Herbst, 1784)	M	P	2	0	0	2
Amara (Amara) aenea (DeGeer, 1774)	M	OM	4	84	20	108
Amara (Amara) familiaris (Duftschmid, 1812)	M	OM	0	0	2	2
Amara (Zezea) fulvipes (Audinet-Serville, 1821)	M	OM	20	2	0	22
Amara (Amara) similata (Gyllenhal, 1810)	M	OM	0	1	0	1
Anchomenus (Anchomenus) dorsalis (Pontoppidan, 1763)	M	P	5	9	6	20
Anchomenus (Anchomenus) binotatus (Fabricius, 1787)	M	OM	82	15	48	145
Anisodactylus (Pseudanisodactylus) signatus (Panzer, 1796)	M	OM	2	0	4	6
Bembidion (Bembidion) quadrimaculatum (Linnaeus, 1760)	M	OM	1	0	0	1
Brachinus (Brachinus) elegans (Chaudoir, 1842)	M	P	1	41	2	44
Brachinus (Brachynidius) sclopeta (Fabricius, 1792)	M	P	0	21	0	21
Calathus (Calathus) fuscipes (Goeze, 1777)	M	P	0	4	1	5
Calathus (Neocalathus) melanocephalus (Linnaeus, 1758)	B	P	0	1	2	3
Calosoma (Campalita) auropunctatum (Herbst, 1784)	M	P	0	6	1	7
Carabus (Carabus) granulatus (Linnaeus, 1758)	D	P	14	91	7	112
Chlaenius (Chlaeniellus) nitidulus (Schrank, 1781)	M	P	35	0	2	37
Clivina (Clivina) fossor (Linnaeus, 1758)	M	P	8	6	9	23
*Diachromus germanus* (Linnaeus, 1758)	M	PHY	0	140	1	141
*Dolichus halensis* (Schaller, 1783)	M	P	0	2	0	2
*Egodroma marginatus* (Dejean, 1829)	M	P	0	0	1	1
Harpalus (Harpalus) affinis (Schrank, 1781)	M	P	16	84	497	597
Harpalus (Harpalus) distinguendus (Duftschmid, 1812)	M	OM	2	5	50	57
Harpalus (Harpalus) oblitus (Dejean, 1829)	M	OM	0	4	1	5
Harpalus (Harpalus) serripes (Quensel in Schonherr, 1806)	M	OM	0	0	1	1
Metallina (Metallina) properans (Stephens, 1828)	M	OM	196	51	383	630
*Microlestes minutulus* (Goeze, 1777)	B	P	0	0	2	2
Panagaeus (Panagaeus) cruxmajor (Linnaeus, 1758)	M	P	6	0	0	6
Parophonus (Ophonomimus) hirsutulus (Dejean, 1829)	M	P	0	1	9	10
Parophonus (Parophonus) maculicornis (Duftschmid, 1812)	M	OM	0	1	0	1
Poecilus (Poecilus) cupreus (Linnaeus, 1758)	M	OM	22	203	81	306
Poecilus (Poecilus) versicolor (Sturm, 1824)	M	OM	2	11	12	25
Pseudoophonus (Pseudoophonus) griseus (Panzer, 1796)	M	P	0	9	70	79
Pseudoophonus (Pseudoophonus) rufipes (De Geer, 1774)	M	OM	76	883	915	1874
Pterostichus (Melanius) aterrimus (Herbst, 1784)	M	OM	0	0	1	1
Pterostichus (Morphnosoma) melanarius (Illiger, 1798)	M	P	0	19	12	31
Pterostichus (Platysma) niger (Schaller, 1783)	M	P	0	1	1	2
Pterostichus (Phonias) strenuus (Panzer, 1796)	M	P	25	19	1	45
Pterostichus (Argutor) vernalis (Panzer, 1796)	M	P	6	10	2	18
*Sphaerotachys hoemorrhoidalis* (Ponza, 1805)	M	P	1	0	0	1
Stenolophus (Stenolophus) teutonus (Schrank, 1781)	M	OM	6	36	10	52
**TOTAL**			534	1758	2157	4449

**Table 3.** List of spiders collected in the three *marcita* fields of the study area during the summer of 2014.

**Family**	**Species**	**Field**			**Total**
		**Casterno**	**Tre Colombaie**	**Sforzesca**	
Gnaphosidae	*Micaria pulicaria* (Sundevall, 1831)	1	0	0	1
Gnaphosidae	*Zelotes longipes* (Koch, 1866)	0	1	1	2
Linyphiidae	*Araeoncus humilis* (Blackwall, 1841)	1	6	0	7
Linyphiidae	*Bathyphantes gracilis* (Blackwall, 1841)	3	3	6	12
Linyphiidae	*Ceratinella brevipes* (Westring, 1851)	2	0	0	2
Linyphiidae	*Diplostyla concolor* (Wider, 1834)	1	7	0	8
Linyphiidae	*Erigone dentipalpis* (Wider, 1834)	11	23	36	70
Linyphiidae	*Gnathonarium dentatum* (Wider, 1834)	4	14	14	32
Linyphiidae	*Mermessus trilobatus* (Emerton, 1882)	2	3	0	5
Linyphiidae	*Microlinyphia pusilla* (Sundevall, 1830)	1	0	0	1
Linyphiidae	*Neriene clathrata* (Sundevall, 1830)	1	0	0	1
Linyphiidae	*Oedothorax apicatus* (Blackwall, 1850)	95	56	75	226
Linyphiidae	*Palliduphantes pallidus* (O. Pickard-Cambridge, 1871)	0	1	0	1
Linyphiidae	*Prinerigone vagans* (Audouin, 1826)	2	12	5	19
Lycosidae	*Alopecosa pulverulenta* (Clerck, 1757)	0	1	1	2
Lycosidae	*Arctosa leopardus* (Sundevall, 1833)	92	43	53	188
Lycosidae	*Pardosa agrestis* (Westring, 1861)	1	0	0	1
Lycosidae	*Pardosa cribrata* (Simon, 1876)	1	0	5	6
Lycosidae	*Pardosa prativaga* (L. Koch, 1870)	12	13	21	46
Lycosidae	*Pardosa proxima* s.l.* (C. L. Koch, 1847)	205	303	223	731
Lycosidae	*Pirata tenuitarsis* (Simon, 1876)	1	0	0	1
Lycosidae	*Piratula hygrophila* (Thorell, 1872)	3	0	0	3
Lycosidae	*Trochosa ruricola* (De Geer, 1778)	37	8	9	48
Tetragnathidae	*Pachygnatha clercki* (Sundevall, 1823)	1	2	6	9
Tetragnathidae	*Pachygnatha degeeri* (Sundevall, 1830)	240	4	7	251
Thomisidae	*Ozyptila sanctuaria* (O. Pickard-Cambridge, 1871)	0	1	0	1
Thomisidae	*Ozyptila simplex* (O. Pickard-Cambridge, 1862)	9	1	0	10
Thomisidae	*Xysticus gallicus* (Simon, 1875)	2	0	0	2
Thomisidae	*Xysticus kochi* (Thorell, 1872)	6	0	0	6
**Total**		**734**	**502**	**462**	**1698**

*taxonomic identification of this species is prior to the new classification from Pantini and Isaia (2019)Isaia et al. (2018)

**Table 4.** List of rove beetle species collected in the three *marcita* fields of the study area during the summer of 2014.

**Species**	**Field**			**Total**
	**Casterno**	**Sforzesca**	**Tre Colombaie**	
Aleochara (Coprophara) bipustulata (Linnaeus, 1760)	8	0	0	8
*Anotylus rugosus* (Fabricius, 1775)	2	0	0	2
*Arpedium quadrum* (Gravenhorst, 1806)	1	0	0	1
*Astrapaeus ulmi* (Rossi, 1790)	0	0	1	1
Atheta (Atheta) aeneicollis (Sharp, 1869)	50	3	76	129
*Atheta fungi* (Gravenhorst, 1806)	0	13	0	13
Atheta (Dimetrotina) laticollis (Stephens, 1832)	0	6	0	6
Atheta (Atheta) triangulum (Kraatz, 1856)	0	7	0	7
Carpelimus (Taenosoma) corticinus (Gravenhorst, 1806)	0	1	0	1
*Cordalia obscura* (Gravenhorst, 1802)	0	4	0	4
*Dinaraea angustula* (Gyllenhal, 1810)	0	4	0	4
Drusilla (Drusilla) canaliculata (Fabricius, 1787)	6	0	0	6
*Euryalea murina* (Erichson, 1839)	5	0	0	5
*Falagria sulcatula* (Gravenhorst, 1806)	1	0	0	1
*Falagrioma thoracica* (Stephens, 1832)	0	0	1	1
*Gabrius* sp. (Stephens, 1829)	0	0	1	1
*Ochthephilum brevipenne* (Mulsant & Rey, 1861)	0	0	1	1
Ocypus (Matidus) brunnipes (Fabricius, 1781)	1	0	0	1
Ocypus (Ocypus) olens (O.F.Müller, 1764)	2	3	5	10
*Omalium caesum* (Gravenhorst, 1806)	6	1	6	13
*Omalium oxyacanthae* (Gravenhorst, 1806)	0	3	4	7
Oxypoda (Oxypoda) opaca (Gravenhorst, 1802)	1	4	3	8
Paederus (Heteropaederus) fuscipes (Curtis, 1826)	10	7	38	55
Philonthus (Philonthus) carbonarius (Gravenhorst, 1802)	6	70	96	172
Philonthus (Philonthus) cognatus (Stephens, 1832)	1	73	3	77
Philonthus (Philonthus) succicola (C.G.Thomson, 1860)	0	1	2	3
Platystethus (Craetopycrus) burlei (Brisout de Barneville, 1862)	0	0	1	1
Platystethus (Craetopycrus) nitens (C. Sahlberg, 1832)	0	0	1	1
*Proteinus ovalis* (Stephens, 1834)	0	0	2	2
*Quedius nitipennis* (Stephens, 1833)	1	0	1	2
Rugilus (Rugilus) orbiculatus (Paykull, 1789)	0	1	3	4
Tachinus (Tachinus) corticinus (Gravenhorst, 1802)	0	7	1	8
Xantholinus (Xantholinus) linearis (Olivier, 1795)	0	0	1	1
Xantholinus (Xantholinus) longiventris (Heer, 1839)	23	1	9	33
**TOTAL**	**124**	**209**	**256**	**589**

**Table 5.** List of butterflies collected in the three *marcita* fields of the study area during the summer of 2014.

**Family**	**Species**	**Field**			**Total**
		**Casterno**	**Tre Colombaie**	**Sforzesca**	
Carabidae	*Loxostege sticticalis* (Linnaeus, 1761)	1	0	0	1
Carabidae	*Pyrausta despicata* (Scopoli, 1763)	0	1	0	1
Geometridae	*Idaea deversaria* (Herrich-Schäffer, 1847)	1	2	0	3
Geometridae	*Timandra comae* (Schmidt, 1931)	0	1	1	2
Hesperiidae	*Pyrgus* sp. (Hübner, 1819)	0	1	0	1
Lycaenidae	*Lycaena dispar* (Haworth, 1802)	1	1	2*	4
Lycaenidae	*Lycaena phlaeas* (Linnaeus, 1761)	0	0	1	1
Noctuidae	*Autographa gamma* (Linnaeus, 1758)	0	2	0	2
Noctuidae	*Rivula sericealis* (Scopoli, 1763)	1	1	0	2
Nymphalidae	*Inachis io* (Linnaeus, 1758)	0	0	4	4
Nymphalidae	*Vanessa cardui* (Linnaeus, 1758)	0	1	0	1
Pieridae	*Colias alfacariensis* (Ribbe, 1905)	1	0	0	1
Pieridae	*Colias crocea* (Fourcroy, 1785)	1	3	1	5
Pieridae	*Pieris rapae* (Linnaeus, 1758)	2	1	5	8
Satyridae	*Coenonympha pamphilus* (Linnaeus, 1758)	3	3	1	7
Satyridae	*Maniola jurtina* (Linnaeus, 1758)	1	0	1	2
**Total**		**12**	**17**	**16**	**45**

*the species was also collected once in 2015 at the field “Amerio 2"

**Table 6.** List of the overwintering beetles collected in the six *marcita* fields of the study area during the winter of 2014-2015. For ground beetles species, information is reported for the wing development WD (M = Macropterous, winged species; D = dimorphic, a species that can be either winged or not winged; B = Brachypterous, species without wings or reduced ones) and diet D (P = Predator, OM = Omnivorous).

**Family**	**Species**	**WD**	**D**	**Field**						**Total**
				**Casterno**	**Tre Colombaie**	**Sforzesca**	**Amerio1**	**Amerio2**	**Garlaschè**	
Carabidae	Agonum (Olisares) emarginatum (Gyllenhal, 1827)	M	P	0	1	0	0	0	0	1
Carabidae	Agonum (Olisares) sexpunctatum (Linnaeus, 1758)	M	P	3	0	0	0	0	0	3
Carabidae	Anchomenus (Anchomenus) dorsalis (Pontoppidan, 1763)	M	P	24	0	0	0	0	0	24
Carabidae	Anisodactylus (Anisodactylus) binotatus (Fabricius, 1787)	M	OM	2	0	0	0	0	0	2
Carabidae	Badister (Badister) bullatus (Schrank, 1798)	M	P	0	3	0	0	0	0	3
Carabidae	Carabus (Carabus) granulatus (Linnaeus, 1758)	D	P	21	7	15	11	3	6	63
Carabidae	Limodromus (Limodromus) assimilis (Paykull, 1790)	M	P	0	0	0	0	3	0	3
Carabidae	Limodromus (Limodromus) krynickii (Sperk, 1835)	M		1	0	9	0	0	0	10
Carabidae	Panagaeus (Panagaeus) cruxmajor (Linnaeus, 1758)	M	P	39	0	0	0	0	0	39
Carabidae	*Paranchus albipes* (Fabricius, 1796)	M		15	1	0	0	0	0	16
Carabidae	Parophonus (Ophonomimus) hirsutulus (Dejean, 1829)	M	OM	1	0	0	0	0	0	1
Carabidae	Pseudoophonus (Pseudoophonus) griseus (Panzer, 1796)	M	OM	1	0	0	0	0	0	1
Carabidae	Pseudoophonus (Pseudoophonus) rufipes (De Geer, 1774)	M	OM	0	0	0	0	0	1	1
Carabidae	Pterostichus (Melanius) aterrimus (Herbst, 1784)	M	P	0	1	0	0	0	0	1
Carabidae	Pterostichus (Phonias) strenuus (Panzer, 1796)	M	P	0	0	0	0	0	2	2
Carabidae	Pterostichus (Argutor) vernalis (Panzer, 1796)	M	P	0	0	20	0	0	0	20
Carabidae	Stenolophus (Stenolophus) teutonus (Schrank, 1781)	M	OM	2	0	2	0	10	0	14
Lucanidae	*Dorcus parallelepipedus* (Linnaeus, 1785)			0	2	0	0	0	0	2
Staphilinidae	Ocypus (Ocypus) olens (O.F.Müller, 1764)		P	0	2	0	0	0	0	2
Staphilinidae	Paederus (Heteropaederus) fuscipes (Curtis, 1826)		P	400	7	0	0	0	1	408
Staphilinidae	*Quedius cruentus* (Olivier, 1795)			0	1	0	0	0	0	1
Staphilinidae	Quedius (Raphirus) nitipennis (Stephens, 1833)			1	0	0	0	0	0	1
**Total**				**510**	**14**	**124**	**11**	**28**	**12**	**618**

**Table 7.** List of grasshoppers and crickets collected in the six *marcita* fields of the study area during summer 2015. For each species, information is reported about the mobility class (Mob) and the habitat specificity class (HS). Mobility classes are: 1 = Sedentary; 2 = Intermediate dispersers; 3 = Mobile species. Habitat specificity classes are: 1 = Generalists; 2 = Medium specialised species; 3 = Specialists (* indicates hygrophilous species).

**Family**	**Species**	**Mob**	**HS**	**Field**						**Total**
				**Amerio 1**	**Amerio 2**	**Casterno**	**Garlaschè**	**Sforzesca**	**Tre Colombaie**	
Acrididae	*Acrida ungarica mediterranea* (Herbst, 1786)	2	1					1		1
Acrididae	*Aiolopus strepens strepens* (Latreille, 1804)	3	2						1	1
Acrididae	*Aiolopus thalassinus thalassinus* (Fabricius, 1781)	3	2	8	1	9	12	8	10	48
Acrididae	*Calliptamus italicus italicus* (Linnaeus, 1758)	2	2	1		2	1	4		8
Acrididae	Chorthippus (Glyptobothrus) brunneus brunneus (Thunberg, 1815)	2	1			3			11	14
Acrididae	Chorthippus (Chorthippus) dorsatus dorsatus (Zetterstedt, 1821)	3	1		1	2		2		5
Acrididae	*Chorthippus* sp. (Fieber, 1852)					2			5	7
Acrididae	Chrysocharon dispar dispar* (Germar, 1834)	2	2				1			1
Acrididae	*Euchorthippus declivus* (Brisout de Barneville, 1848)	3	2		8	1	2	7	19	37
Acrididae	*Glyptobothrus* sp. (Fieber, 1852)			3	1	2	2	4	1	13
Acrididae	*Locusta migratoria cinerascens* (Linnaeus, 1758)	3	2	1			1	1	3	6
Acrididae	*Mecostethus parapleurus parapleurus* (Hagenbach, 1822)	2	2	5	3	2			1	11
Acrididae	*Oedipoda caerulescens caerulescens* (Linnaeus, 1758)	3	2	1						1
Acrididae	Omocestus (Omocestus) rufipes (Zetterstedt, 1821)	2	2	6		2	1	2	6	17
Acrididae	*Pezotettix giornae* (Rossi, 1794)	3	1						1	1
Acrididae	*Pseudochorthippus parallelus parallelus* (Zetterstedt, 1821)	3	3	12	5	15	2	10	11	55
Tetrigidae	*Tetrix subulata* (Linnaeus, 1758)	3	3			1	1	1	1	4
Tetrigidae	*Tetrix tenuicornis* (Sahlberg, 1893)	2	3			1				1
Tetrigidae	*Tetrix* sp. (Latreille, 1802)					1				1
Tettigonidae	*Platycleis grisea grisea* (Fabricius, 1781)	2	1					1		1
Tettigonidae	Roeseliana azami azami* (Finot, 1892)	1	2	1						1
Tettigonidae	*Ruspolia nitidula* (Scopoli, 1786)	3	3	3	2	4	6	4	7	26
Tettigonidae	*Tettigonia viridissima* (Linnaeus, 1758)	3	3				1			1
Tettigonidae	*Tettigonia* sp. (Linnaeus, 1758)						1			1
**Total**				**41**	**21**	**47**	**31**	**45**	**77**	**26**

**Table 8.** Species richness and diversity of ground beetles during summer 2014. The Table shows the observed diversity, the asymptotic estimates (Estimator), the estimated bootstrap S.E. and 95% confidence intervals (LCL and UCL) for Hill numbers of order q (0: Species richness, 1: Shannon diversity and 2: Simpson diversity).

**Sites**	**Observed diversity**		**Estimator**	**S.E.**	**LCL**	**UCL**
Casterno	Species richness	24	27.119	3.652	24.505	43.269
	Shannon diversity	8.56	8.793	0.477	8.56	9.728
	Simpson diversity	5.248	5.29	0.353	5.248	5.981
Tre Colombaie	Species richness	31	36.331	4.927	32.143	55.87
	Shannon diversity	5.544	5.595	0.137	5.544	5.863
	Simpson diversity	3.727	3.732	0.086	3.727	3.901
Sforzesca	Species richness	29	37.995	10.168	30.519	82.248
	Shannon diversity	6.753	6.829	0.239	6.753	7.297
	Simpson diversity	3.451	3.455	0.129	3.451	3.708

**Table 9.** Results from permutational multivariate analysis of variance for differences in species composition amongst the fields, based on a Bray-Curtis resemblance matrix with *P*-values obtained by 9999 permutations.

**Source**	**df**	**SS**	**MS**	**Pseudo-F**	**P(perm)**	**Unique perms**
Ground beetles	2	13709	6854.4	12.635	**0.0001**	9906
Spiders	2	5442.8	2721.4	4.926	**0.001**	9998
Rove beetles	2	15186	7593.1	5.1917	**0.0001**	9920

SS, sum of squares; MS, mean sum of squares; Pseudo-*F*, *F* value by permutation. Bold indicates statistical significance at *P <* 0.05.

**Table 10.** Results from permutational multivariate analysis of variance pairwise tests for differences in species composition between pairs of fields.

**Taxa**	**Fields**	**t**	**Unique perm**	**P(MC)**
Ground beetles	Casterno, Tre colombaie	3.3453	424	**0.001**
	Casterno, Sforzesca	3.5711	413	**0.001**
	Tre colombaie, Sforzesca	3.9663	409	**0.001**
Spiders	Casterno, Tre Colombaie	2.5317	400	**0.002**
	Casterno, Sforzesca	2.4161	413	**0.004**
	Tre Colombaie, Sforzesca	1.4634	409	0.06
Rove beetles	Casterno, Tre Colombaie	1.4314	407	0.082
	Casterno, Sforzesca	2.4808	407	**0.003**
	Tre Colombaie, Sforzesca	2.2146	409	**0.008**

The significantly different species composition is indicated by bold *p*-values obtained by permutation.

**Table 11.** Results from SIMPER analysis for differences in species composition amongst the three *marcita* fields. This analysis shows which species contribute the most to dissimilarity between pairs of fields and provides a percentage of this contribution in a decreasing dissimilarity order.

**Taxa**	**Species**	% **Dissimilarity**	**Average abundance**		**CONTRIB**%	**CUM**%
			**Casterno**	**Sforzesca**		
Ground Beetles	Pseudoophonus (Pseudoophonus) rufipes (De Geer, 1774)	64.19	3.39	12.3	19.1	19.1
	Harpalus (Harpalus) affinis (Schrank, 1781)		1.1	9.04	17.1	36.2
Spiders	*Pachygnatha degeeri* (Sundevall, 1830)	44.42	6.2	0.87	21.7	21.7
	*Oedothorax apicatus* (Blackwall, 1850)		3.37	3.36	8.39	30.09
Rove Beetles	Philonthus (Philonthus) cognatus (Stephens, 1832)	81.20	0.17	3.04	15.77	15.77
	Philonthus (Philonthus) carbonarius (Gravenhorst, 1802)		0.80	3.30	13.64	29.41
			**Tre Colombaie**	**Sforzesca**		
Ground Beetle	Harpalus (Harpalus) affinis (Schrank, 1781)	43.32	9.04	3.44	11.99	11.99
	Metallina (Metallina) properans (Stephens, 1828)		7.93	2.76	10.95	22.93
Spiders	*Erigone dentipalpis* (Wider, 1834)	33.83	1.54	2.15	9.14	9.14
	*Pardosa proxima* s.l* (C. L. Koch, 1847)		7.09	6.01	8.31	17.44
Rove Beetles	Philonthus (Philonthus) cognatus (Stephens, 1832)	61.95	0.40	3.04	15.54	15.54
	Atheta (Atheta) aeneicollis (Sharp, 1869)		2.89	0.29	15.34	30.88
			**Casterno**	**Tre Colombaie**		
Ground Beetle	Pseudoophonus (Pseudoophonus) rufipes (De Geer, 1774)	58.75	3.39	12.3	19.1	19.1
	Harpalus (Harpalus) affinis (Schrank, 1781)		1.1	9.04	17.1	36.2
Spiders	*Pachygnatha degeeri* (Sundevall, 1830)	46.39	6.2	0.57	21.42	21.42
	*Oedothorax apicatus* (Blackwall, 1850)		3.37	3.01	7.61	29.03
Rove Beetles	Philonthus (Philonthus) carbonarius (Gravenhorst, 1802)	63.99	0.80	3.91	19.94	19.94
	Atheta (Atheta) aeneicollis (Sharp, 1869)		2.21	2.89	15.08	35.03

**Table 12.** Species richness and diversity of spiders during summer 2014. The Table shows the observed diversity, the asymptotic estimates (Estimator), the estimated bootstrap S.E. and 95% confidence intervals (LCL and UCL) for Hill numbers of order q (0: Species richness, 1: Shannon diversity and 2: Simpson diversity).

**Sites**	**Observed diversity**		**Estimator**	**s.e.**	**LCL**	**UCL**
Casterno	Species richness	26	36.111	9.006	28.261	71.221
	Shannon diversity	6.42	6.591	0.309	6.42	7.197
	Simpson diversity	4.542	4.565	0.183	4.542	4.924
Tre Colombaie	Species richness	19	31.475	17.106	20.662	112.652
	Shannon diversity	4.697	4.838	0.327	4.697	5.48
	Simpson diversity	2.571	2.579	0.17	2.571	2.912
Sforzesca	Species richness	15	17.994	4.532	15.352	40.431
	Shannon diversity	5.611	5.723	0.312	5.611	6.334
	Simpson diversity	3.538	3.557	0.227	3.538	4.002

**Table 13.** Species richness and diversity of rove beetles during summer 2014. The Table shows the observed diversity, the asymptotic estimates (Estimator), the estimated bootstrap S.E. and 95% confidence intervals (LCL and UCL) for Hill numbers of order q (0: Species richness, 1: Shannon diversity and 2: Simpson diversity).

**Sites**	**Observed diversity**		**Estimator**	**s.e.**	**LCL**	**UCL**
Casterno	Species richness	16	24.927	10.095	17.507	68.87
	Shannon diversity	7.345	8.142	0.915	7.345	9.936
	Simpson diversity	4.604	4.743	0.662	4.604	6.039
Tre Colombaie	Species richness	21	41.171	20.111	24.959	123.771
	Shannon diversity	5.947	6.424	0.616	5.947	7.63
	Simpson diversity	3.924	3.97	0.253	3.924	4.466
Sforzesca	Species richness	18	27.948	10.229	19.883	70.563
	Shannon diversity	7.009	7.522	0.786	7.009	9.062
	Simpson diversity	4.339	4.415	0.436	4.339	5.27
